# Baricitinib-associated changes in global gene expression during a 24-week phase II clinical systemic lupus erythematosus trial implicates a mechanism of action through multiple immune-related pathways

**DOI:** 10.1136/lupus-2020-000424

**Published:** 2020-10-09

**Authors:** Thomas Dörner, Yoshiya Tanaka, Michelle A Petri, Josef S Smolen, Daniel J Wallace, Ernst R Dow, Richard E Higgs, Guilherme Rocha, Brenda Crowe, Robert J Benschop, Nicole L Byers, Maria E Silk, Stephanie de Bono, Damiano Fantini, Robert W Hoffman

**Affiliations:** 1 DRFZ Berlin and Department of Rheumatology and Clinical Immunology, Charite University Hospital Berlin, Berlin, Germany; 2 The First Department of Internal Medicine, School of Medicine, University of Occupational & Environmental Health, Kitakyushu, Japan; 3 Division of Rheumatology, Johns Hopkins University School of Medicine, Baltimore, Maryland, USA; 4 Division of Rheumatology, Medical University of Vienna, Wien, Austria; 5 Department of Rheumatology, Cedars-Sinai Medical Center, West Hollywood, California, USA; 6 Eli Lilly and Company, Indianapolis, Indiana, USA

**Keywords:** cytokines, lupus erythematosus, systemic, therapeutics, autoimmune diseases

## Abstract

**Objective:**

To characterise the molecular pathways impacted by the pharmacologic effects of the Janus kinase (JAK) 1 and JAK2 inhibitor baricitinib in SLE.

**Methods:**

In a phase II, 24-week, randomised, placebo-controlled, double-blind study (JAHH), RNA was isolated from whole blood in 274 patients and analysed using Affymetrix HTA2.0 array. Serum cytokines were measured using ultrasensitive quantitative assays.

**Results:**

Gene expression profiling demonstrated an elevation of *STAT1*, *STAT2* and multiple interferon (IFN) responsive genes at baseline in patients with SLE. Statistical and gene network analyses demonstrated that baricitinib treatment reduced the mRNA expression of functionally interconnected genes involved in SLE including *STAT1*-target, *STAT2*-target and *STAT4-*target genes and multiple IFN responsive genes. At baseline, serum cytokines IFN-α, IFN-γ, interleukin (IL)-12p40 and IL-6 were measurable and elevated above healthy controls. Treatment with baricitinib significantly decreased serum IL-12p40 and IL-6 cytokine levels at week 12, which persisted through week 24.

**Conclusion:**

Baricitinib treatment induced significant reduction in the RNA expression of a network of genes associated with the JAK/STAT pathway, cytokine signalling and SLE pathogenesis. Baricitinib consistently reduced serum levels of two key cytokines implicated in SLE pathogenesis, IL-12p40 and IL-6.

## Introduction

SLE is an autoimmune disease that is characterised by systemic inflammation in multiple organs.^1 2^ Despite improvements in therapy, substantial unmet medical need exists in SLE. A plethora of cytokines have been implicated in the pathogenesis of SLE, including an excess of interferons (IFNs), B cell activating factor (BAFF), a proliferation inducing ligand, interleukin (IL)-6, IL-12, IL-17, IL-23, tumour necrosis factor, as well as deficiency of IL-2 and IL-10.^2^ Treatments are under study that target these cytokines, such as type I IFN, to potentially mitigate against SLE pathogenesis.

Many studies have reported elevation of IFN in SLE either measured directly or more often indirectly through elevation of IFN response gene mRNA expression.^3 4^ Several anti-IFN-specific monoclonal antibodies have been studied in clinical trials to determine their efficacy and safety in SLE. Among these, rontalizumab was not efficacious in treating active SLE,^5^ and only a modest clinical effect was observed in a trial testing another anti-IFN mAb: sifalizumab.^6^ In a phase II trial of a mAb targeting the IFN-α receptor, which blocks all type I IFN species, the mAb anifrolumab met the study primary endpoint of Systemic Lupus Responder Index (SRI)-4 at week 24 with a sustained reduction in oral corticosteroids from weeks 12 to 24.^7 8^ Subsequently, two phase III clinical trials, TULIP 1 and TULIP 2, have been completed using anifrolumab,^9 10^ while one met the primary endpoint of British Isles Clinical Lupus Activity index, a second trial failed to meet its primary endpoint of the SRI-4 response.

In addition to targeting IFNs, a proof of concept study targeting IL-6 has reported potential efficacy,^11^ and in a phase II study targeting the p40 subunit shared by IL-12 and IL-23, patients treated with mAb ustekinumab 10 mg had a higher response rate according to SRI-4 than patients in the placebo group.^12^ Nevertheless, a phase III study of ustekinumab has recently been discontinued.^13^ These findings support the concept that multiple cytokines such as IFN, IL-6 and the IL-12p40 pathway may have a role in SLE pathogenesis and could be viable targets for drug therapy.

Baricitinib is approved for the treatment of moderate-to-severe active rheumatoid arthritis in adults in over 65 countries including the USA, Japan and countries in the European Union. Baricitinib inhibits Janus kinase (JAK) 1 and JAK2 signalling^13^ via STAT1 and STAT2 pathways, which may impact the release of several proinflammatory cytokines, including type I IFNs, IFN-γ, IL-6, IL-12, IL-23 and others.^14 15^ In a phase II study of baricitinib, patients with SLE showed improvement in arthritis or rash at week 24, as well as SRI-4 and other secondary endpoints.^16^ Baricitinib, therefore, has the potential to simultaneously affect several key cytokines implicated in the pathogenesis of SLE.^2 15^ The goal of the present study was to examine the gene signature profiles and serum cytokines of SLE patients who were treated with baricitinib and to characterise molecular and cellular immune pathways impacted by baricitinib.

## Patients and methods

### Patient characteristics and study design

SLE patient samples were obtained from the double-blind, randomised, placebo-controlled, 24-week phase II clinical trial, labelled JAHH.^16^ Patients were ≥18 years of age and had a diagnosis of SLE. At baseline, patients were required to have a positive ANA or a positive antidouble stranded DNA (anti-dsDNA), a clinical Systemic Lupus Erythematosus Disease Activity Index-2000 (SLEDAI-2K) of 4 or greater and arthritis or rash as defined by the SLEDAI-2K. Study drug was added to existing stable background standard of care therapy. Prednisone or equivalent was limited to a maximum daily dose of 20 mg at study entry and could not be increased after randomisation. Stable doses of non-steroidal anti-inflammatory drugs, a single immunosuppressant (azathioprine, methotrexate or mycophenolate mofetil) or a single antimalarial were allowed. Active severe lupus nephritis or active CNS lupus were not permitted. Patients were randomly assigned to receive once-daily baricitinib 2 mg, baricitinib 4 mg or placebo for 24 weeks. The primary endpoint of the study was the proportion of patients achieving resolution of arthritis or rash as defined by the SLEDAI-2K at week 24.^16^ All patients and control subjects provided written informed consent. Full details of the JAHH clinical trial have been published.^16^

### RNA isolation, analysis and hybridisation

Whole blood was collected in PAX gene tubes (Thermo Fisher Scientific) at baseline and weeks 2, 4, 12 and 24. Total RNA was isolated, and RNA quality was assessed postextraction as previously described.^3^ Complementary DNA (cDNA) was prepared as previously described, and labelled cDNA was hybridised to the GeneChip Human Transcriptome Array 2.0 (HTA2.0) according to the manufacturer’s instructions.^3^

### Microarray analysis

Microarray analyses were performed on the Affymetrix HTA2.0 array and analysed as recently reported.^3^ Comparison of patients with SLE with healthy controls was performed with preprocessed array data from a combined data set of ILLUMINATE-1 and ILLUMINATE-2, which were two phase III studies of the anti-BAFF targeting mAb tabalumab compared with healthy controls.^3^ Analyses from study JAHH were performed with and without correction for total cell count and are shown using corrected counts.

### Microarray preprocessing and normalisation

HTA2.0 microarray preprocessing was performed by summarising probe-level microarray data to the ‘transcript cluster’ (TC) level using TCs as defined by Affymetrix. Background correction and quantile normalisation were carried out using standard robust multiarray average.^17^ Summarisation to the TC level was done by estimating sample effects in an analysis of variance model with sample and probe effects using robust regression (rlm function in the R MASS package).

### Cytokine expression analysis

JAHH serum samples were analysed for IL-2, IL-3, IL-5, IL-6, IL-10, IL-17A, IL-12p40, IL-12p70, GM-CSF and IFN-γ using a Meso Scale Multiplex cytokine panel and ultrasensitive quantitative assays (Quanterix SiMoA platform).^18^ JAHH plasma samples were analysed for IL-21 and IFN-α. Cytokine detection was performed using ultrasensitive quantitative assays.^19^ Cytokine concentrations were log-transformed before computing expression changes from baseline. Samples with undetectable analyte levels (concentration below lower limit of quantification (LLOQ)) were imputed to 0.5 × LLOQ. Patients with undetectable cytokine levels at both baseline and week 12 were removed from the analysis.

### Gene network analysis

Genes included in this analysis were selected from the list of differentially expressed genes for the baricitinib 4 mg dose at week 12 compared with placebo. The genes were selected by two methods: (1) the 50 genes most significantly changed with baricitinib treatment and (2) genes that interact with JAK1 or JAK2 via transcriptional regulation or phosphorylation as defined by the curated MetaBase (Clarivate) database and had an adjusted p<0.05. In order to graphically show the interactions, these genes, along with *STAT1*, *STAT2*, *JAK1*, *JAK2* and *TYK2*, were queried against the known interactions in MetaBase, and genes with known interactions were connected and displayed using Cytoscape; genes that were not directly connected to this network are not shown.

### N-of-1 single subject analyses

Unpaired single-subject analyses were conducted similar to that of Gardeux *et al*.^20^ Briefly, two gene signatures were defined including JAK1-activated genes or JAK1/JAK2/TYK2-activated genes, according to the gene network information from the MetaBase (Clarivate) database. Baseline expression of all signature genes was analysed from each SLE patient and compared with the mean expression from the patient population by Wilcoxon test.^20^ Subjects showing statistically elevated or reduced expression of the signature at baseline were included in signature-high and signature-low groups, respectively. For each patient and for each visit, the change in SLEDAI-2K from baseline was computed. Next, for each signature group (high and low), the average SLEDAI-2K change was computed at each visit. Significance was assessed by running Monte Carlo simulations with 10 000 iterations. At each iteration, baseline signature status was randomly shuffled, and average SLEDAI-2K changes for each group and at each visit were computed. Statistical significance was determined by computing the overall difference between average SLEDAI-2K changes from the signature-high and signature-low groups, and then assessing it against the distribution from the Monte Carlo simulations.

### Statistical analysis

All variables analysed were quantitative even if there was a categorical version of the variable. Pharmacological analysis was based on microarray data and performed by first fitting a mixed-effect, repeated measures model within each TC separately. The response variable was log2-expression. Main effects, including treatment and time along with their interactions, were included in the model as fixed discrete effects. Age, sex and cell counts were included in the model as additional control covariates. A spatial correlation structure was used to model the covariance of measurements made on the same patient across different visits/time-points. Pharmacodynamic (PD) effects were estimated based on the difference between the variation in expression for each treatment group at a given time point and baseline and the variation in expression in the placebo group at that same time point and baseline. The MMRM model was fit for each TC separately and comparisons of baricitinib 2 mg and baricitinib 4 mg to placebo were performed at each time point. PD effects were evaluated only on the TCs associated to the top 50 genes according to differences between SLE and controls, and the corrections for multiple comparisons were done by computing FDR adjusted q-values using a Benjamini-Hochberg procedure.^21^

## Results

### Patient disposition

The demographics of the patients are shown in table 1.^16^ Most patients were female with a mean age of 43–46 years and disease duration of 10–12 years. All patients with SLE included in this analysis participated in the phase II trial of baricitinib (JAHH), had given informed consent for genetic studies and had provided adequate amounts of high-quality RNA available, as well as serum and plasma, at the time of testing (n=274). This resulted in a total of 274 patients, compared with the total number of 314 patients enrolled in the trial for RNA and a total of 270 patients for cytokine testing.

**Table 1:** Baseline characteristics and disease activity*

	Placebo (n=90)	Baricitinib 2 mg (n=92)	Baricitinib 4 mg (n=92)
Age, years, mean (SD)	45.5 (12.6)	43.4 (11.0)	46.2 (12.0)
Female, n (%)	85 (94.4)	83 (90.2)	88 (95.7)
Time since onset of SLE, years, mean (SD)	10.2 (8.0)	11.8 (9.4)	12.1 (10.4)
SLEDAI-2K score, mean (SD)	8.9 (2.9)	8.6 (3.2)	8.8 (3.1)
SLEDAI-2K≥10, n (%)	39 (43.3)	28 (30.4)	37 (40.2)
SLEDAI-2K organ system involvement, n (%)			
Musculoskeletal	79 (87.8)	82 (89.1)	87 (94.6)
Mucocutaneous	79 (87.8)	73 (79.3)	81 (88.0)
Immunological	53 (58.9)	53 (57.6)	57 (62.0)
Haematological	12 (13.3)	7 (7.6)	3 (3.3)
Renal	8 (8.9)	8 (8.7)	4 (4.3)
Vascular	1 (1.1)	3 (3.3)	3 (3.3)
CNS	2 (2.2)	0	2 (2.2)
Cardiovascular and respiratory	2 (2.2)	1 (1.1)	1 (1.1)
Constitutional	1 (1.1)	2 (2.2)	0
≥1A or ≥2B BILAG scores, n (%)	52 (57.8)	49 (53.3)	62 (67.4)
Physician’s Global Assessment, mean (SD)	48.7 (16.9)	48.7 (16.1)	51.9 (16.1)
CLASI activity score, mean (SD)	5.2 (5.9)	4.0 (5.7)	3.9 (3.4)
Tender joint count, mean (SD)	7.7 (5.9)	8.6 (6.5)	8.6 (5.9)
Swollen joint count, mean (SD)	5.4 (4.7)	5.2 (4.6)	5.8 (4.2)
Urine protein:creatinine ratio, n (%)			
≤50 mg/mmol	85 (94.4)	89 (96.7)	86 (93.5)
˃50 mg/mmol	5 (5.6)	3 (3.3)	6 (6.5)
eGFR (MDRD), mL/min/1.73 m2, mean (SD)	92.5 (22.8)	96.0 (21.7)	91.1 (24.2)
SLICC/ACR Damage Index score, mean (SD)	0.6 (1.0)	0.4 (0.6)	0.4 (0.9)
Haematological, mean (SD)			
Haemoglobin, mmol/L-Fe	8.0 (0.9)	8.1 (0.9)	7.9 (0.9)
Platelets, 10^9^/L	239.7 (65.5)	250.0 (77.4)	245.6 (72.7)
Leucocytes, 10^9^/L	5.5 (2.1)	6.2 (2.6)	5.9 (2.4)
Neutrophils, 10^9^/L	3.7 (1.7)	4.3 (2.2)	4.0 (2.1)
Lymphocytes, 10^9^/L	1.3 (0.6)	1.4 (0.7)	1.4 (0.7)
Monocytes, 10^9^/L	0.3 (0.1)	0.3 (0.1)	0.3 (0.1)
Immunologic			
ANA titre ≥1:80, n (%)	89 (98.9)	91 (98.9)	87 (94.6)
Anti-dsDNA, IU/mL, mean (SD)	83.9 (133.6)	115.5 (190.3)	140.1 (300.1)
Anti-dsDNA ≥30 IU/mL, n (%)	42 (46.7)	47 (51.1)	46 (50.0)
C3, g/L, mean (SD)	1.1 (0.3)	1.1 (0.3)	1.1 (0.3)
C3 <90 mg/dL, n (%)	24 (26.7)	24 (26.1)	28 (30.4)
C4, g/L, mean (SD)	0.2 (0.1)	0.2 (0.1)	0.2 (0.1)
C4 <10 mg/dL, n (%)	11 (12.2)	22 (23.9)	13 (14.1)
Anti-Sm ≥30 AU/mL, n (%)	9 (10.8)	5 (6.0)	6 (7.2)
Anti-RNP ≥30 AU/mL, n (%)	24 (28.9)	18 (21.4)	24 (28.9)
Anti-SSA/Ro 52≥30 AU/mL, n (%)	28 (33.7)	22 (26.2)	24 (28.9)
Anti-SSA/Ro 60≥30 AU/mL, n (%)	45 (54.2)	36 (42.9)	34 (41.0)
Anti-SSB/LA≥30 AU/mL, n (%)	13 (15.7)	12 (14.3)	13 (15.7)
Antiphospholipid positive, n (%)	16 (19.0)	23 (27.1)	19 (23.2)
Anticardiolipin IgG ˃14 GPL, n (%)	6 (7.1)	6 (7.1)	4 (4.9)
Anticardiolipin IgM ˃12 MPL, n (%)	13 (15.5)	20 (23.5)	18 (22.0)
Serum IgG, g/L, mean (SD)	14.1 (6.3)	13.9 (3.7)	13.6 (4.9)
Serum IgM, g/L, mean (SD)	1.1 (1.7)	1.0 (0.6)	1.0 (0.7)
Concomitant medications			
Corticosteroids, n (%)	66 (73.3)	70 (76.1)	66 (71.7)
Prednisone dose (or equivalent; of those taking corticosteroids), mg/day, mean (SD)	7.9 (4.6)	8.4 (5.7)	8.5 (4.4)
Prednisone dose (or equivalent; of those taking corticosteroids), ≥7.5 mg/day, n (%)	31 (47.0)	33 (47.1)	35 (53.0)
Antimalarials, n (%)	65 (72.2)	61 (66.3)	67 (72.8)
Immunosuppressants, n (%)	39 (43.3)	41 (44.6)	43 (46.7)
MTX	11 (12.2)	15 (16.3)	13 (14.1)
AZA	13 (14.4)	8 (8.7)	9 (9.8)
MMF	10 (11.1)	8 (8.7)	14 (15.2)
NSAID, n (%)	24 (26.7)	25 (27.2)	27 (29.3)

*For patients included in the gene expression analyses.

ACR, American College of Rheumatology; AZA, azathioprine; BILAG, British Isles Lupus Assessment Group; C, complement; CLASI, Cutaneous Lupus Erythematosus Disease Area and Severity Index; CNS, central nervous system; dsDNA, double-stranded DNA; eGFR, estimated glomerular filtration rate; Ig, immunoglobulin; MDRD, Modification of Diet in Renal Disease Study equation; MMF, mycophenolate mofetil; mmol/L-Fe, mmol/L (Fe); MTX, methotrexate; n, number of patients in the specified category; N, number of patients in analysis population; NSAID, nonsteroidal anti-inflammatory drug; RNP, ribonucleoprotein; SLEDAI-2K, Systemic Lupus Erythematosus Disease Activity Index-2000; SLICC, Systemic Lupus International Collaborating Clinics.

### Baseline gene expression in SLE

**Figure 1:**
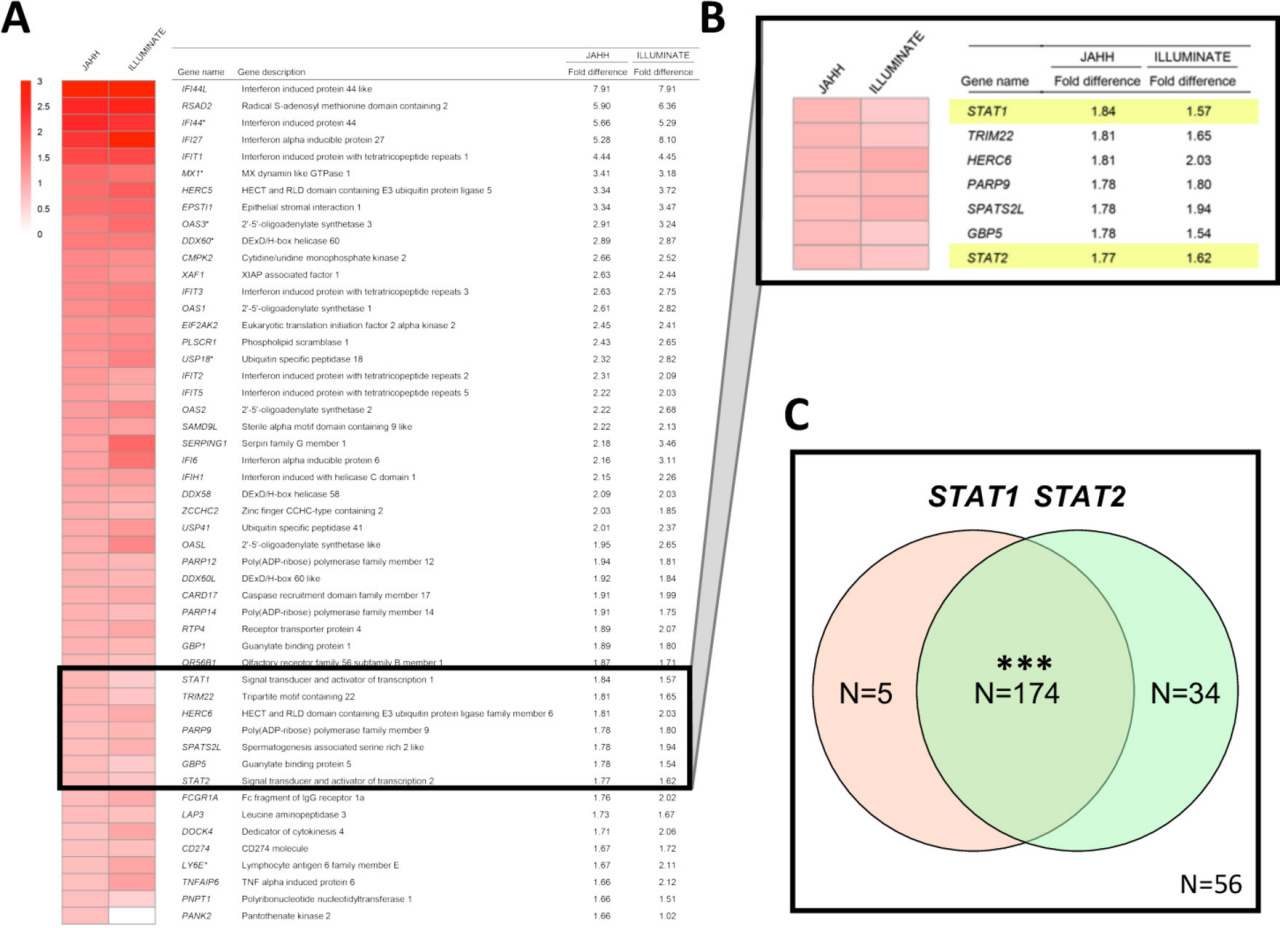
(A) The top 50 genes upregulated (ranked from high to low) based on the difference in expression between patients with SLE) from JAHH and healthy control subjects are shown. Gene names were obtained from the MetaBase (Clarivate) database. Findings were compared with those from the ILLUMINATE study, analysed using data deposited in the NCBI Geo database (GSE88887), where an independent group of controls was compared with ILLUMINATE patients with SLE at baseline and expression fold differences are shown. Results shown for patients with SLE from JAHH compared with healthy controls had q-values <2.7×10^–8^. (B) Exploded heatmap showing the top 36th to 42nd top DE genes, which included STAT1 and STAT2. (C) Coordinated mRNA overexpression of signal transducer and activator of transcription (STAT) 1 and STAT2 in patients with SLE. Patients were assigned to the STAT1 high and the STAT2 high groups if their STAT1 and STAT2 mRNA expression at baseline was higher than the 95th percentile of healthy controls. The number of patients in the STAT1 high (orange circle) and STAT2 high (green circle) groups are indicated in the Venn diagram. A total number of 56 patients had normal expression of both STAT1 and STAT2. A total of five patients showed high expression of STAT1 but not STAT2, while 34 patients had increased STAT2 and normal STAT1 expression. A total of 174 patients had increased expression of both STAT1 and STAT2. Asterisks indicate statistically significant association (***= p<0.001). DE, differentially expressed.

A comparison yielding a ranked list of the top 50 genes elevated in patients with active SLE at trial enrolment compared with healthy blood donor controls is shown in figure 1A. An extended list of the top 150 genes elevated and a list of the top 150 genes decreased compared with controls is shown in online supplementary tables S1 and S2, respectively. As described in figure 1, there were genes with large fold increases comparing patients to healthy controls in JAHH at baseline, and these were highly statistically significant (online supplementary table S1). These findings were compared with those from the ILLUMINATE study where an independent group of controls was compared with 1760 patients with SLE.^3^ At baseline, findings from ILLUMINATE were similar to JAHH both in terms of the rank order of genes and the fold changes that are detected (figure 1A). Many of these genes have been defined functionally as IFN responsive genes. Importantly, the *STAT1* (figure 1B) gene exhibited significantly elevated expression at baseline among the SLE patients who all had active disease at study entry. Furthermore, among patients with SLE, but not healthy controls, there was significant overexpression of paired *STAT1* with *STAT2* at baseline (p<2.0×10^−16^) (figure 1C) consistent with the predominance of IFNs.

10.1136/lupus-2020-000424.supp1 Supplementary data

### Baricitinib induces change in gene expression

**Figure 2:**
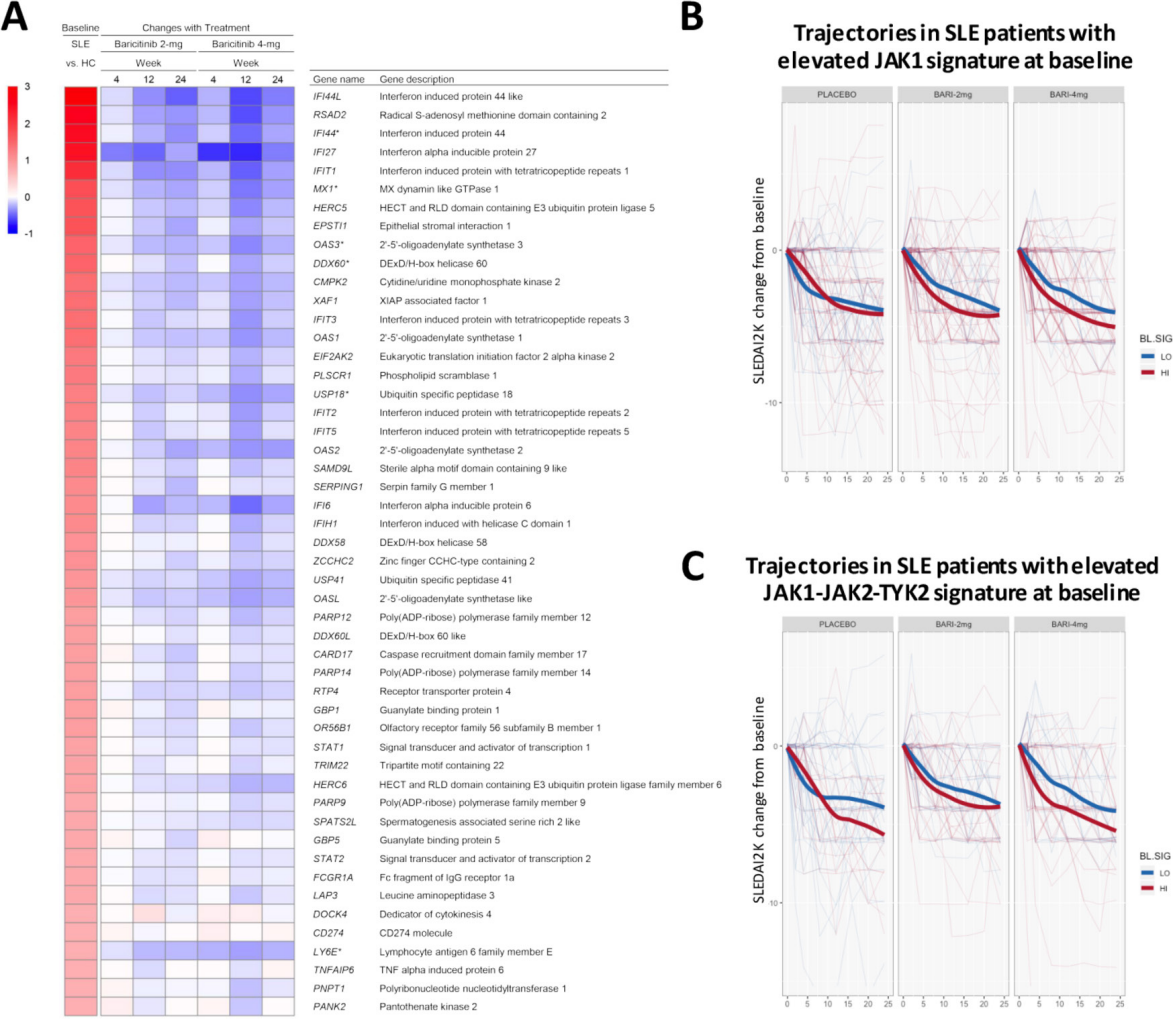
(A) Heatmap of top 50 genes upregulated in patients with SLE (ranked from high to low) showing the difference in expression between patients with SLE and healthy control subjects (far left column) and the corresponding gene expression changes in patients with SLE treated with baricitinib 2 mg and 4 mg at weeks 4, 12 and 24 (right columns) as compared with placebo. Gene name was defined using the MetaBase (Clarivate) database. (B and C) Changes in SLEDAI-2K from baseline for SLE patients with elevated (red) or reduced (blue) expression of the JAK1 (B) or JAK1-JAK2-TYK2 (C) gene signature at baseline. Thin lines correspond to individual patients. Thick lines show the LOESS-smoothed population average by group. Monte Carlo simulations were performed to assess if signature-high averages were overall lower than signature-low averages across visits and by baricitinib treatment group (B, JAK1-signature: placebo, p=0.80; baricitinib 2 mg, p=0.02; baricitinib 4 mg, p=0.0003. C, JAK1/JAK2/TYK2-signature: placebo, p=0.07; baricitinib 2 mg, p=0.07; baricitinib 4 mg, p=0.0002). JAK, Janus kinase; SLEDAI-2K, Systemic Lupus Erythematosus Disease Activity Index-2000.

For genes that were upregulated, baricitinib treatment induced statistically significant changes in gene expression, which was most notable with the 4 mg dose (figure 2). The heatmap in figure 2A shows the top 50 genes with elevated expression in SLE versus controls, ranked by fold change. The subsequent columns show changes for baricitinib 2 mg and 4 mg treated patients at weeks 4, 12 and 24 compared with baseline. The heatmap demonstrates the quantitative reduction in gene expression with baricitinib treatment for many of the genes elevated in SLE versus controls, with many IFN responsive genes represented among the top 50. Consistent with the phosphorylation-mediated mechanism of STAT1 activation,^22^ there was no statistically significant downregulation of *STAT1* RNA in samples from baricitinib-treated patients (figure 2A and online supplementary table S3).

The effect of baricitinib on the JAK/STAT signalling pathway and the association with SLEDAI-2K was further investigated. At week 0, patients with elevated (signature-high) or reduced (signature-low) expression of JAK1-activated genes were identified using an N-of-1-pathway single-subject analysis. Next, changes in the SLEDAI-2K were calculated from baseline for each patient in the signature-high group versus signature-low group across the three treatment arms. SLEDAI-2K trajectories were aggregated according to the baseline grouping (signature high vs low groups) and smoothed by LOESS. This analysis (figure 2B and C) revealed that patients with a relative elevation of JAK1-target genes at baseline had an overall better response to treatment with baricitinib 4 mg. Interestingly, patients from the placebo group exhibited overlapping SLEDAI-2K changes over time independent of the relative expression of the JAK1 signature at baseline (week 0). Similar results were obtained when baseline patients were grouped based on an expanded list of genes, including targets of JAK1, JAK2 and TYK2 (figure 2C), but the placebo response in this analysis was higher after week 12. These results are consistent with the observation that multiple JAK/STAT-target genes showed changes on treatment that correlated with changes in SLEDAI-2K (online supplementary table S4).

### Gene network analysis

**Figure 3:**
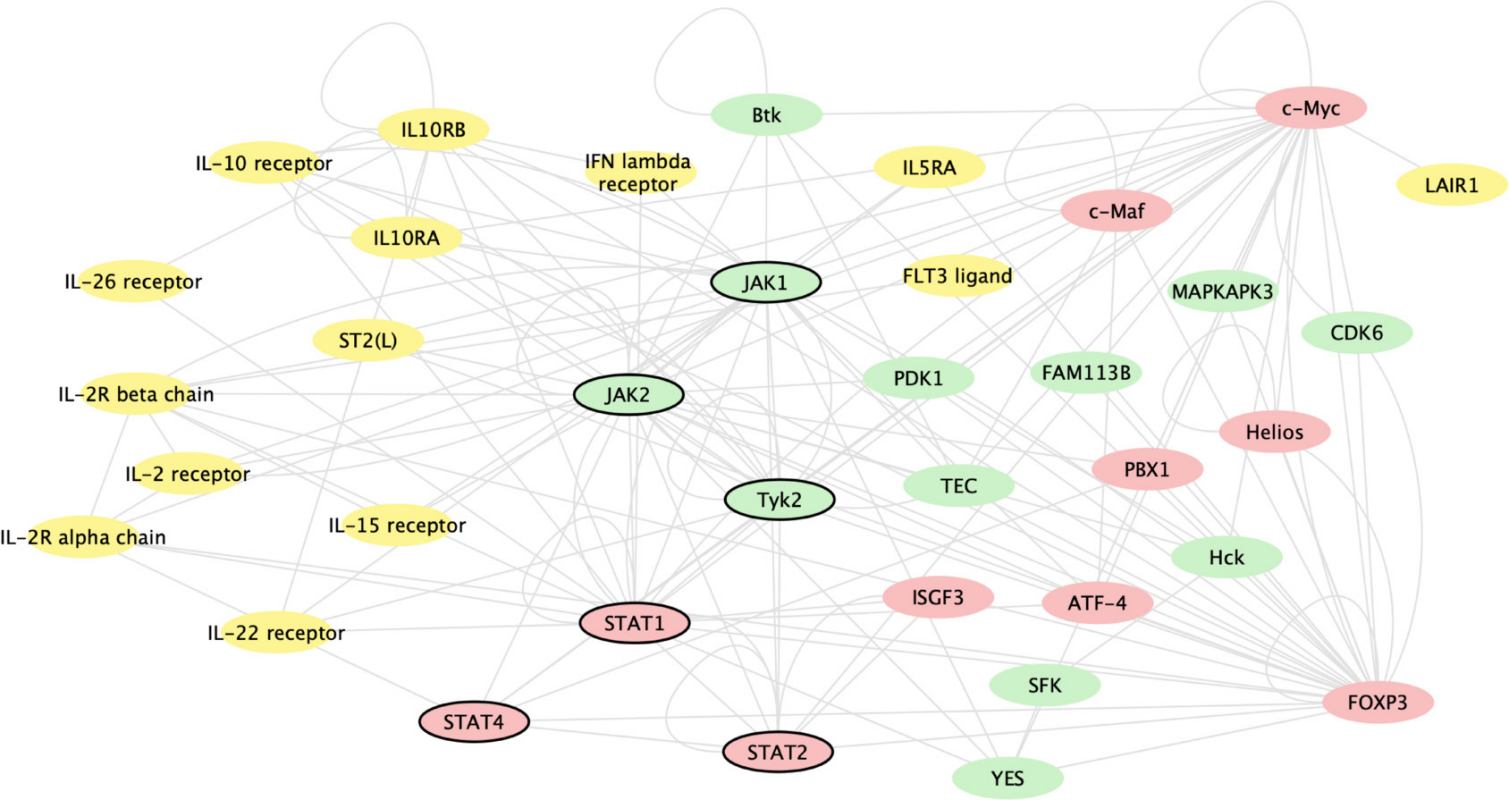
Gene network analysis of genes changed based on baricitinib’s inhibition of JAK1 and JAK2 signalling and the most significantly baricitinib-induced changed genes, regardless of mechanism. The analysis includes genes changed with baricitinib 4 mg at week 12 compared with placebo. The genes included were identified by two methods: (1) the 50 genes most significantly changed with baricitinib treatment and (2) genes that interact with JAK1 or JAK2 via transcriptional regulation or phosphorylation as defined by the curated MetaBase (Calarivate) database and had an adjusted p<0.05. In order to graphically show the interactions, these genes, along with STAT1, STAT2, JAK1, JAK2 and Tyk2, were queried against the known interactions in MetaBase and kinases (green), ligands/receptors (yellow) and transcription factors (red) with known interactions were connected and displayed using cytoscape (cytoscape.org); genes that were not directly connected to this network or were in other categories are not shown. Genes of key interest in the network are indicated by bold outline, including STAT1, STAT2, STAT4, JAK1, JAK2 and Tyk2. JAK, Janus kinase.

Baricitinib induced changes in gene expression, regardless of mechanism, by inhibiting JAK1 and JAK2 signalling. Figure 3 shows genes whose expression changed based on baricitinib’s inhibition of JAK1 and JAK2 signalling and the most significantly baricitinib-induced changed genes. Baricitinib altered the expression of genes involved in immune pathways, including multiple genes for key cytokines (*STAT* 1, 2 and 4), cytokine receptors, T cells, regulatory cells and cytokine regulators that are associated with the immune pathogenesis of SLE.

### Serum cytokine expression

**Figure 4:**
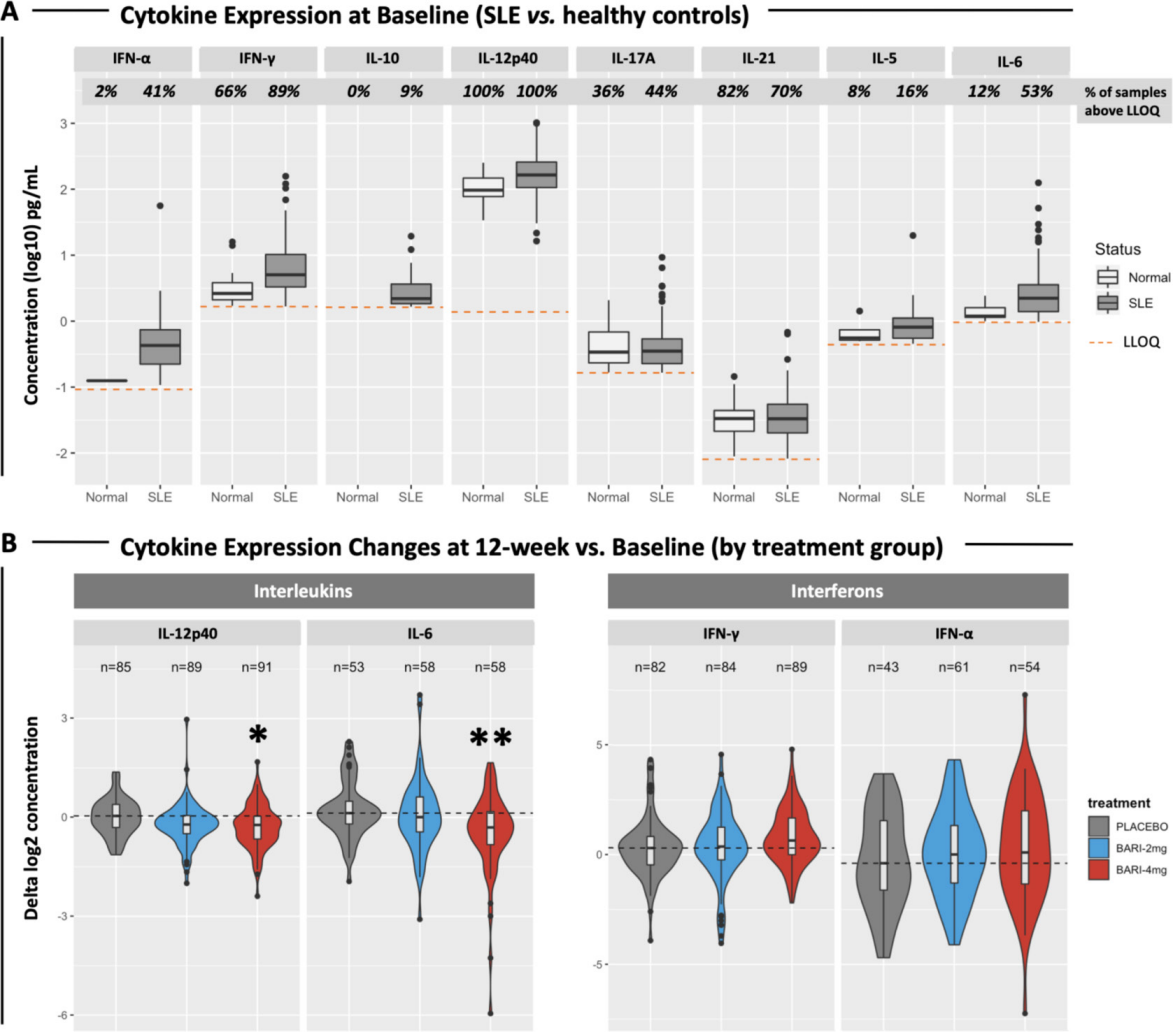
(A) Boxplots showing the expression levels of SLE cytokines in 270 patients at baseline and in 50 healthy controls. Percentages of samples with detectable cytokine levels are reported. Dashed lines indicate the LLOQ for each cytokine. The following cytokines were significantly elevated in SLE samples compared with controls: IFN-α (p=1.95×10^−7^), IFN-γ (p=1.45×10^–7^), IL-12p40 (p=2.20×10^–13^), IL-6 (p=2.84×10^–7^). (B) Violin plots showing changes (deltas) in cytokine expression at week 12 compared with baseline. Patients with undetectable levels at both baseline and week 12 were removed from the analysis. The number of subjects included in the analysis is reported. Violin colours indicate treatment groups. Dashed lines indicate the median cytokine expression change detected from baseline in the placebo group. Asterisks indicate statistically significant changes (*=p<0.05; **=p<0.01). IL, interleukin; LLOQ, lower limit of quantification.

At baseline, expression levels of many cytokines were detectable in a small fraction of healthy control samples, although some cytokines, such as INF-γ, IL-12p40 and IL-17A and IL-21, were routinely detected (figure 4A). The fraction of SLE patients with detectable cytokine levels was higher than controls for IFN-α, IFN-γ, IL-10, IL-5, IL-6 and IL-12p40, and the mean serum concentrations were higher in SLE (figure 4A).

Cytokine levels were analysed for changes in the serum of JAHH patients by treatment arm (figure 4B). There were significant changes in IL-12p40 and IL-6, whose protein levels were reduced in the baricitinib 4 mg treatment arm but not in the placebo arm (figure 4B). The baricitinib-induced reduction of IL-12p40 (p=0.016) and IL-6 (p=0.001) was most pronounced at 12 weeks of treatment (figure 4B). A similar trend was observed also after 24 weeks of treatment (not shown), but the changes did not meet statistical significance.

## Discussion

The goal of this study was to characterise the molecular pathways impacted by the pharmacological effects of the JAK1 and JAK2 inhibitor baricitinib in SLE using RNA and serum samples from a phase II study of baricitinib that successfully met its primary endpoint. Baseline gene expression profiling of patients demonstrated that there was an elevation of *STAT1*, *STAT2* and multiple IFN responsive genes, when compared with healthy controls. Baricitinib treatment reduced the mRNA expression of functionally interconnected genes involved in SLE including *STAT1*-target, *STAT2-*target and *STAT4-*target genes and multiple IFN responsive genes.^2^ Furthermore, the study demonstrated that treatment with baricitinib 4 mg significantly decreased serum IL-12p40 and IL-6 cytokine levels at week 12, which persisted through week 24. Thus, treatment with baricitinib 4 mg induced significant reduction in the RNA expression of a network of genes associated with the JAK/STAT pathway, cytokine signalling and SLE pathogenesis, and it consistently reduced serum levels of two key disease-associated cytokines, IL-12p40 and IL-6. In this regard, a *JAK1*-associated gene profile has been identified correlating with treatment-associated changes found for the serum cytokine IL-6 and IL-12p40 responses, which will require further validation/replication. Notably, other cytokine signalling can be influenced indirectly by complex interaction of signalling networks that interface with *JAK/STAT*.

The demographics of patients with SLE in the phase II study JAHH were similar to other recent phase II and III clinical trials suggesting that the baseline findings here may be broadly applicable.^3 4^ The baseline gene expression profiles of the patients studied yielded findings similar to the much larger 1760 patient ILLUMINATE studies, as well as other smaller studies, where large fold differences from healthy controls in expression of type I IFN responsive genes were observed in 50%–75% of patients.^3 4^

A key finding of the present study was the identification of baseline elevations of *STAT1, STAT2* and *STAT4*, and/or downstream genes linked to the JAK/STAT pathway. The observations on *STAT1, STAT* 2 and *STAT4* are distinctive to this study and again highlight the known importance of these genes and their associated signalling pathways in SLE.^22 23^

A second important finding of the present study was that pharmacologically induced changes in gene expression following baricitinib therapy resulted in significant reduction in *STAT1*-targets. Of note, patients with a relative elevation in the expression of JAK1 pathway genes at baseline displayed a better response to baricitinib 4 mg according to changes in SLEDAI-2K (figure 2B) or SRI-4 (data not shown) over 24 weeks. While it was anticipated that baricitinib could reduce JAK/STAT targets expression, this is the first publication to directly demonstrate this in a clinical trial linking such changes to clinical improvement.

Gene network analysis revealed further that there were expression changes in genes that are involved directly in classical, canonical JAK/STAT signal transduction, including *STAT1* and *STAT4*, where genetic polymorphisms have been identified as a risk factor for both disease susceptibility and severity in SLE.^23 24^

This study is among the largest published surveys that includes both whole blood RNA expression and serum cytokine levels in SLE.^3 4 25 26^ While gene expression studies allow for a comprehensive survey of cellular and molecular immune processes, it is an indirect measure of circulating serum cytokines. Based on precedents in the medical literature, the state of our current understanding of the pathogenesis of SLE and technical feasibility, the study attempted to measure a select number of key cytokines using serum or plasma samples from JAHH. These included: IFN-α, IFN-γ, IL-10, IL-12p40, IL-17A, IL-5 and IL-6 levels. Overall, with few exceptions, the cytokines tested were elevated in patients with SLE at baseline as compared with healthy controls. These results strongly support the hypothesis that SLE is not a single cytokine-driven disease but is characterised by global dysregulation of the cytokine signalling network.^2^ Therefore, a pharmacological approach targeting multiple cytokines at the same time, such as the inhibition of the JAK/STAT axis, could be highly beneficial in the treatment of SLE. In further support of this, it was found that baricitinib treatment was associated with statistically significant decreases of serum IL-12p40 and IL-6, supporting the concept that reducing the levels of these cytokines may be effective in treating SLE, as was suggested by previous clinical trials targeting these molecules.^11 12^ Various transcription factors have been implicated in the regulation of IL-6 and IL-12p40 expression, including AP-1, C/EBP and nuclear factor-κB but not the JAK/STAT axis. Therefore, the molecular mechanisms responsible for the baricitinib-induced reduction of cytokine expression are still unclear and may entail cross-talk between STATs and other transcription factors, or suppressive effects on the proliferation and survival of cytokine-producing cells. Changes of *STAT*4 possibly linked to IL-12p40 under baricitinib 4 mg provided some confirmation between transcriptional changes and reduced cytokines, which was not seen for STAT3 connected to IL-6. Although differences between transcriptional changes and related protein expression can be found, the overall findings clearly indicate comprehensive inhibition of inflammatory pathways.

In conclusion, the mechanism of action of baricitinib in SLE may be mediated through the inhibition of multiple immune-related genes and cytokines. The study findings confirm that the JAK/STAT pathways appear to have a central role in the pharmacological effect of baricitinib in SLE. Baricitinib induced changes in the expression of *STAT1*/*STAT2* target genes, and these were associated with treatment response. Serum cytokine measurement support the hypothesis that IL-12p40 and IL-6 may have a role in SLE pathogenesis and contribute to the PD effect of baricitinib. Finally, the two ongoing phase III studies of baricitinib in SLE will help to further define the clinical benefits of baricitinib in SLE and the molecular mechanisms of its action.^27 28^

## References

[1] Tsokos GC, Lo MS, Costa Reis P, et al New insights into the immunopathogenesis of systemic lupus erythematosus. Nat Rev Rheumatol 2016;12:716–30. 10.1038/nrrheum.2016.186 27872476

[2] Wahren-Herlenius M, Dörner T Immunopathogenic mechanisms of systemic autoimmune disease. Lancet 2013;382:819–31. 10.1016/S0140-6736(13)60954-X 23993191

[3] Hoffman RW, Merrill JT, Alarcón-Riquelme MME, et al Gene expression and pharmacodynamic changes in 1,760 systemic lupus erythematosus patients from two phase III trials of BAFF blockade with tabalumab. Arthritis Rheumatol 2017;69:643–54. 10.1002/art.39950 27723281 PMC6585752

[4] Crow MK, Olferiev M, Kirou KA Type I interferons in autoimmune disease. Annu Rev Pathol 2019;14:369–93. 10.1146/annurev-pathol-020117-043952 30332560

[5] Kalunian KC, Merrill JT, Maciuca R, et al A phase II study of the efficacy and safety of rontalizumab (rhuMAb interferon-α) in patients with systemic lupus erythematosus (rose). Ann Rheum Dis 2016;75:196–202. 10.1136/annrheumdis-2014-206090 26038091

[6] Khamashta M, Merrill JT, Werth VP, et al Sifalimumab, an anti-interferon-α monoclonal antibody, in moderate to severe systemic lupus erythematosus: a randomised, double-blind, placebo-controlled study. Ann Rheum Dis 2016;75:1909–16. 10.1136/annrheumdis-2015-208562 27009916 PMC5099191

[7] Furie R, Khamashta M, Merrill JT, et al Anifrolumab, an Anti-Interferon-α receptor monoclonal antibody, in moderate-to-severe systemic lupus erythematosus. Arthritis Rheumatol 2017;69:376–86. 10.1002/art.39962 28130918 PMC5299497

[8] AstraZeneca Update on tulip 1 phase III trial for anifrolumab in systemic lupus erythematosus, 2018.

[9] Furie RA, Morand EF, Bruce IN, et al Type I interferon inhibitor anifrolumab in active systemic lupus erythematosus (TULIP-1): a randomised, controlled, phase 3 trial. Lancet Rheumatol 2019;1:e208–19. 10.1016/S2665-9913(19)30076-1 38229377

[10] Morand EF, Furie R, Tanaka Y, et al Trial of Anifrolumab in active systemic lupus erythematosus. N Engl J Med 2020;382:211–21. 10.1056/NEJMoa1912196 31851795

[11] Wallace DJ, Strand V, Merrill JT, et al Efficacy and safety of an interleukin 6 monoclonal antibody for the treatment of systemic lupus erythematosus: a phase II dose-ranging randomised controlled trial. Ann Rheum Dis 2017;76:534–42. 10.1136/annrheumdis-2016-209668 27672124 PMC5446001

[12] van Vollenhoven RF, Hahn BH, Tsokos GC, et al Efficacy and safety of ustekinumab, an IL-12 and IL-23 inhibitor, in patients with active systemic lupus erythematosus: results of a multicentre, double-blind, phase 2, randomised, controlled study. The Lancet 2018;392:1330–9. 10.1016/S0140-6736(18)32167-6 30249507

[13] Fridman JS, Scherle PA, Collins R, et al Selective inhibition of JAK1 and JAK2 is efficacious in rodent models of arthritis: preclinical characterization of INCB028050. J.i. 2010;184:5298–307. 10.4049/jimmunol.0902819 20363976

[14] Schwartz DM, Bonelli M, Gadina M, et al Type I/II cytokines, JAKs, and new strategies for treating autoimmune diseases. Nat Rev Rheumatol 2016;12:25–36. 10.1038/nrrheum.2015.167 26633291 PMC4688091

[15] Schwartz DM, Kanno Y, Villarino A, et al Jak inhibition as a therapeutic strategy for immune and inflammatory diseases. Nat Rev Drug Discov 2017;16:843–62. 10.1038/nrd.2017.201 29104284

[16] Wallace DJ, Furie RA, Tanaka Y, et al Baricitinib for systemic lupus erythematosus: a double-blind, randomised, placebo-controlled, phase 2 trial. The Lancet 2018;392:222–31. 10.1016/S0140-6736(18)31363-1 30043749

[17] Irizarry RA, Bolstad BM, Collin F, et al Summaries of Affymetrix GeneChip probe level data. Nucleic Acids Res 2003;31:15e–15. 10.1093/nar/gng015 PMC150247 12582260

[18] Poorbaugh J, Samanta T, Bright SW, et al Measurement of IL-21 in human serum and plasma using ultrasensitive MSD S-PLEX® and Quanterix SiMoA methodologies. J Immunol Methods 2019;466:9–16. 10.1016/j.jim.2018.12.005 30590020

[19] Rodero MP, Decalf J, Bondet V, et al Detection of interferon alpha protein reveals differential levels and cellular sources in disease. J Exp Med 2017;214:1547–55. 10.1084/jem.20161451 28420733 PMC5413335

[20] Gardeux V, Achour I, Li J, et al ‘N-of-1- pathways ’ unveils personal deregulated mechanisms from a single pair of RNA-Seq samples: towards precision medicine. J Am Med Inform Assoc 2014;21:1015–25. 10.1136/amiajnl-2013-002519 25301808 PMC4215042

[21] Leon AC, Heo M Sample sizes required to detect interactions between two binary fixed-effects in a mixed-effects linear regression model. Comput Stat Data Anal 2009;53:603–8. 10.1016/j.csda.2008.06.010 20084090 PMC2678722

[22] Rawlings JS, Rosler KM, Harrison DA The JAK/STAT signaling pathway. J Cell Sci 2004;117:1281–3. 10.1242/jcs.00963 15020666

[23] Bolin K, Sandling JK, Zickert A, et al Association of STAT4 polymorphism with severe renal insufficiency in lupus nephritis. PLoS One 2013;8:e84450 10.1371/journal.pone.0084450 24386384 PMC3873995

[24] Svenungsson E, Gustafsson J, Leonard D, et al A STAT4 risk allele is associated with ischaemic cerebrovascular events and anti-phospholipid antibodies in systemic lupus erythematosus. Ann Rheum Dis 2010;69:834–40. 10.1136/ard.2009.115535 19762360

[25] Mok CC The Jakinibs in systemic lupus erythematosus: progress and prospects. Expert Opin Investig Drugs 2019;28:85–92. 10.1080/13543784.2019.1551358 30462559

[26] Reynolds JA, McCarthy EM, Haque S, et al Cytokine profiling in active and quiescent SLE reveals distinct patient subpopulations. Arthritis Res Ther 2018;20:173. 10.1186/s13075-018-1666-0 30092845 PMC6085716

[27] ClinicalTrials.gov A study of Baricitinib (LY3009104) in participants with systemic lupus erythematosus (brave I) (NCT03616912), 2018 Available: https://clinicaltrials.gov/ct2/show/NCT03616912?term=baricitinib&cond=SLE&rank=4

[28] ClinicalTrials.gov A study of Baricitinib in participants with systemic lupus erythematosus (brave II) (NCT03616964), 2018 Available: https://clinicaltrials.gov/ct2/show/NCT03616964?term=baricitinib&cond=SLE&rank=3

