# Supplementary material for: Baricitinib-associated changes in global gene expression during a 24-week phase II clinical systemic lupus erythematosus trial implicates a mechanism of action through multiple immune-related pathways
Source: Lupus Sci Med. 2020 Oct 9;7(1):e000424. doi: 10.1136/lupus-2020-000424 (PMC7549481; doi:10.1136/lupus-2020-000424)
Supplement: Supplementary data [file lupus-2020-000424supp001.pdf]

**SUPPLEMENTARY FILE**
**Supplementary Tables**

**Table S1.** Baseline gene expression in SLE patients versus healthy controls, including upregulated genes with the greatest fold change from JAHH*

| Gene name | Gene description | JAHH Fold difference | JAHH q value | ILLUMINATE Fold difference | ILLUMINATE q value |
|---|---|---|---|---|---|
| *IFI44L* | interferon induced protein 44 like | 7.91 | <1.0e-08 | 7.91 | <1.0e-08 |
| *RSAD2* | radical S-adenosyl methionine domain containing 2 | 5.90 | <1.0e-08 | 6.36 | <1.0e-08 |
| *IFI44* | interferon induced protein 44 | 5.66 | <1.0e-08 | 5.29 | <1.0e-08 |
| *IFI27* | interferon alpha inducible protein 27 | 5.28 | <1.0e-08 | 8.10 | <1.0e-08 |
| *IFIT1* | interferon induced protein with tetratricopeptide repeats 1 | 4.44 | <1.0e-08 | 4.45 | <1.0e-08 |
| *MX1* | MX dynamin like GTPase 1 | 3.41 | <1.0e-08 | 3.18 | <1.0e-08 |
| *HERC5* | HECT and RLD domain containing E3 ubiquitin protein ligase 5 | 3.34 | <1.0e-08 | 3.72 | <1.0e-08 |
| *EPSTI1* | epithelial stromal interaction 1 | 3.34 | <1.0e-08 | 3.47 | <1.0e-08 |
| *OAS3* | 2'-5'-oligoadenylate synthetase 3 | 2.91 | <1.0e-08 | 3.24 | <1.0e-08 |
| *DDX60* | DExD/H-box helicase 60 | 2.89 | <1.0e-08 | 2.87 | <1.0e-08 |
| *CMPK2* | cytidine/uridine monophosphate kinase 2 | 2.66 | <1.0e-08 | 2.52 | <1.0e-08 |
| *XAF1* | XIAP associated factor 1 | 2.63 | <1.0e-08 | 2.44 | <1.0e-08 |
| *IFIT3* | interferon induced protein with tetratricopeptide repeats 3 | 2.63 | <1.0e-08 | 2.75 | <1.0e-08 |
| *OAS1* | 2'-5'-oligoadenylate synthetase 1 | 2.61 | <1.0e-08 | 2.82 | <1.0e-08 |
| *EIF2AK2* | eukaryotic translation initiation factor 2 alpha kinase 2 | 2.45 | <1.0e-08 | 2.41 | <1.0e-08 |
| *PLSCR1* | phospholipid scramblase 1 | 2.43 | <1.0e-08 | 2.65 | <1.0e-08 |
| *USP18* | ubiquitin specific peptidase 18 | 2.32 | <1.0e-08 | 2.82 | <1.0e-08 |
| *IFIT2* | interferon induced protein with tetratricopeptide repeats 2 | 2.31 | <1.0e-08 | 2.09 | <1.0e-08 |
| *IFIT5* | interferon induced protein with tetratricopeptide repeats 5 | 2.22 | <1.0e-08 | 2.03 | <1.0e-08 |
| *OAS2* | 2'-5'-oligoadenylate synthetase 2 | 2.22 | <1.0e-08 | 2.68 | <1.0e-08 |
| *SAMD9L* | sterile alpha motif domain containing 9 like | 2.22 | <1.0e-08 | 2.13 | <1.0e-08 |
| *SERPING1* | serpin family G member 1 | 2.18 | 1.50e -08 | 3.46 | <1.0e-08 |

| | | JAHH | | ILLUMINATE | |
|---|---|---|---|---|---|
| Gene name | Gene description | Fold difference | q value | Fold difference | q value |
| *IFI6* | interferon alpha inducible protein 6 | 2.16 | <1.0e-08 | 3.11 | <1.0e-08 |
| *IFIH1* | interferon induced with helicase C domain 1 | 2.15 | <1.0e-08 | 2.26 | <1.0e-08 |
| *DDX58* | DExD/H-box helicase 58 | 2.09 | <1.0e-08 | 2.03 | <1.0e-08 |
| *ZCCHC2* | zinc finger CCHC-type containing 2 | 2.03 | <1.0e-08 | 1.85 | <1.0e-08 |
| *USP41* | ubiquitin specific peptidase 41 | 2.01 | <1.0e-08 | 2.37 | <1.0e-08 |
| *OASL* | 2'-5'-oligoadenylate synthetase like | 1.95 | <1.0e-08 | 2.65 | <1.0e-08 |
| *PARP12* | poly(ADP-ribose) polymerase family member 12 | 1.94 | <1.0e-08 | 1.81 | <1.0e-08 |
| *DDX60L* | DExD/H-box 60 like | 1.92 | <1.0e-08 | 1.84 | <1.0e-08 |
| *CARD17* | caspase recruitment domain family member 17 | 1.91 | <1.0e-08 | 1.99 | <1.0e-08 |
| *PARP14* | poly(ADP-ribose) polymerase family member 14 | 1.91 | <1.0e-08 | 1.75 | <1.0e-08 |
| *RTP4* | receptor transporter protein 4 | 1.89 | <1.0e-08 | 2.07 | <1.0e-08 |
| *GBP1* | guanylate binding protein 1 | 1.89 | <1.0e-08 | 1.80 | <1.0e-08 |
| *OR56B1* | olfactory receptor family 56 subfamily B member 1 | 1.87 | <1.0e-08 | 1.71 | <1.0e-08 |
| *STAT1* | signal transducer and activator of transcription 1 | 1.84 | <1.0e-08 | 1.57 | <1.0e-08 |
| *TRIM22* | tripartite motif containing 22 | 1.81 | <1.0e-08 | 1.65 | <1.0e-08 |
| *HERC6* | HECT and RLD domain containing E3 ubiquitin protein ligase family member 6 | 1.81 | <1.0e-08 | 2.03 | <1.0e-08 |
| *PARP9* | poly(ADP-ribose) polymerase family member 9 | 1.78 | <1.0e-08 | 1.80 | <1.0e-08 |
| *SPATS2L* | spermatogenesis associated serine rich 2 like | 1.78 | <1.0e-08 | 1.94 | <1.0e-08 |
| *GBP5* | guanylate binding protein 5 | 1.78 | <1.0e-08 | 1.54 | 3.30e-06 |
| *STAT2* | signal transducer and activator of transcription 2 | 1.77 | <1.0e-08 | 1.62 | <1.0e-08 |
| *FCGR1A* | Fc fragment of IgG receptor Ia | 1.76 | <1.0e-08 | 2.02 | <1.0e-08 |
| *LAP3* | leucine aminopeptidase 3 | 1.73 | <1.0e-08 | 1.67 | 2.00e-07 |
| *DOCK4* | dedicator of cytokinesis 4 | 1.71 | <1.0e-08 | 2.06 | <1.0e-08 |
| *CD274* | CD274 molecule | 1.67 | <1.0e-08 | 1.72 | 1.50e-08 |
| *LY6E* | lymphocyte antigen 6 family member E | 1.67 | <1.0e-08 | 2.11 | <1.0e-08 |
| *TNFAIP6* | TNF alpha induced protein 6 | 1.66 | 2.70e-08 | 2.12 | <1.0e-08 |
| *PNPT1* | polyribonucleotide | 1.66 | <1.0e-08 | 1.51 | 6.60e-07 |

| | | JAHH | | ILLUMINATE | |
|---|---|---|---|---|---|
| Gene name | Gene description | Fold difference | q value | Fold difference | q value |
| | nucleotidyltransferase 1 | | | | |
| *PANK2* | pantothenate kinase 2 | 1.66 | <1.0e-08 | 1.02 | 8.40e-01 |
| *SAMD9* | sterile alpha motif domain containing 9 | 1.65 | <1.0e-08 | 1.60 | <1.0e-08 |
| *GBP3* | guanylate binding protein 3 | 1.64 | <1.0e-08 | 1.48 | 1.70e-06 |
| *TNFSF13B* | TNF superfamily member 13b | 1.64 | <1.0e-08 | 1.59 | <1.0e-08 |
| *ZBP1* | Z-DNA binding protein 1 | 1.63 | <1.0e-08 | 1.63 | <1.0e-08 |
| *CLEC12A* | C-type lectin domain family 12 member A | 1.62 | 2.50e-06 | 1.17 | 3.90e-01 |
| *SLFN12* | schlafen family member 12 | 1.60 | <1.0e-08 | 1.29 | 3.60e-04 |
| *RNF213* | ring finger protein 213 | 1.60 | <1.0e-08 | 1.53 | <1.0e-08 |
| *BST2* | bone marrow stromal cell antigen 2 | 1.60 | <1.0e-08 | 1.59 | <1.0e-08 |
| *SAT1* | spermidine/spermine N1-acetyltransferase 1 | 1.59 | <1.0e-08 | 1.29 | 3.60e-05 |
| *MX2* | MX dynamin like GTPase 2 | 1.58 | <1.0e-08 | 1.58 | <1.0e-08 |
| *CLEC12B* | C-type lectin domain family 12 member B | 1.57 | 2.60e-08 | 1.21 | 2.50e-01 |
| *SIGLEC1* | sialic acid binding Ig like lectin 1 | 1.56 | <1.0e-08 | 2.01 | <1.0e-08 |
| *HIST2H4B* | histone cluster 2 H4 family member b | 1.56 | <1.0e-08 | 1.15 | 4.20e-02 |
| *WSB1* | WD repeat and SOCS box containing 1 | 1.55 | <1.0e-08 | 1.15 | 1.80e-02 |
| *FFAR2* | free fatty acid receptor 2 | 1.53 | <1.0e-08 | 1.54 | 2.90e-08 |
| *CCR1* | C-C motif chemokine receptor 1 | 1.52 | 1.40e-08 | 1.85 | <1.0e-08 |
| *FAM8A1* | family with sequence similarity 8 member A1 | 1.50 | <1.0e-08 | 1.35 | <1.0e-08 |
| *DHX58* | DExH-box helicase 58 | 1.50 | <1.0e-08 | 1.50 | <1.0e-08 |
| *TDRD7* | tudor domain containing 7 | 1.50 | <1.0e-08 | 1.47 | <1.0e-08 |
| *GPR15* | G protein-coupled receptor 15 | 1.49 | 1.00e-05 | 1.65 | 3.50e-04 |
| *PGAP1* | post-GPI attachment to proteins 1 | 1.49 | <1.0e-08 | 1.36 | 1.90e-06 |
| *TMEM123* | transmembrane protein 123 | 1.49 | <1.0e-08 | 1.37 | 1.80e-07 |
| *IFI16* | interferon gamma inducible protein 16 | 1.49 | <1.0e-08 | 1.55 | <1.0e-08 |
| *ABCA1* | ATP binding cassette subfamily A member 1 | 1.48 | <1.0e-08 | 1.61 | <1.0e-08 |
| *FCGR1B* | Fc fragment of IgG receptor Ib | 1.48 | <1.0e-08 | 1.44 | <1.0e-08 |
| *HIST1H4D* | histone cluster 1 H4 family member d | 1.47 | <1.0e-08 | 1.35 | 3.30e-06 |
| *GK* | glycerol kinase | 1.47 | <1.0e-08 | 1.23 | 1.10e-02 |
| *ACOT9* | acyl-CoA thioesterase 9 | 1.47 | <1.0e-08 | 1.31 | 2.70e-07 |

| | | JAHH | | ILLUMINATE | |
|---|---|---|---|---|---|
| Gene name | Gene description | Fold difference | q value | Fold difference | q value |
| *SP100* | SP100 nuclear antigen | 1.47 | <1.0e-08 | 1.37 | <1.0e-08 |
| *EVI2A* | ecotropic viral integration site 2A | 1.46 | 2.30e-07 | 1.09 | 2.00e-01 |
| *PTEN* | phosphatase and tensin homolog | 1.46 | <1.0e-08 | -1.01 | 9.40e-01 |
| *GBP2* | guanylate binding protein 2 | 1.46 | <1.0e-08 | 1.37 | 8.90e-08 |
| *TRIM38* | tripartite motif containing 38 | 1.46 | <1.0e-08 | 1.30 | 9.40e-07 |
| *NT5C3A* | 5'-nucleotidase, cytosolic IIIA | 1.45 | <1.0e-08 | 1.27 | 1.90e-04 |
| *SP140* | SP140 nuclear body protein | 1.45 | <1.0e-08 | 1.33 | 1.40e-05 |
| *LTB* | lymphotoxin beta | 1.45 | <1.0e-08 | 1.14 | 8.40e-02 |
| *TNFSF10* | TNF superfamily member 10 | 1.45 | <1.0e-08 | 1.42 | <1.0e-08 |
| *NAIP* | NLR family apoptosis inhibitory protein | 1.45 | <1.0e-08 | 1.40 | 1.80e-05 |
| *TAP1* | transporter 1, ATP binding cassette subfamily B member | 1.45 | <1.0e-08 | 1.37 | <1.0e-08 |
| *HIST2H2BF* | histone cluster 2 H2B family member f | 1.44 | <1.0e-08 | 1.43 | 1.50e-06 |
| *PTP4A1* | protein tyrosine phosphatase type IVA, member 1 | 1.44 | <1.0e-08 | 1.40 | 5.10e-08 |
| *CMTR1* | cap methyltransferase 1 | 1.42 | <1.0e-08 | 1.40 | <1.0e-08 |
| *AIM2* | absent in melanoma 2 | 1.42 | <1.0e-08 | 1.47 | 3.00e-08 |
| *FAS* | Fas cell surface death receptor | 1.42 | <1.0e-08 | 1.01 | 9.50e-01 |
| *NABP1* | nucleic acid binding protein 1 | 1.42 | <1.0e-08 | 1.08 | 4.00e-01 |
| *CASP1* | caspase 1 | 1.41 | <1.0e-08 | 1.32 | 3.50e-06 |
| *OTOF* | otoferlin | 1.41 | 2.90e-06 | 1.85 | 1.80e-06 |
| *IFI35* | interferon induced protein 35 | 1.41 | <1.0e-08 | 1.62 | <1.0e-08 |
| *FAM174B* | family with sequence similarity 174 member B | 1.41 | <1.0e-08 | 1.32 | 3.10e-06 |
| *UTS2* | urotensin 2 | 1.41 | 1.80e-03 | 1.41 | 6.30e-02 |
| *ZNF117* | zinc finger protein 117 | 1.40 | 9.10e-06 | 1.03 | 8.40e-01 |
| *CBWD5* | COBW domain containing 5 | 1.40 | <1.0e-08 | 1.12 | 7.20e-02 |
| *SLC26A8* | solute carrier family 26 member 8 | 1.40 | <1.0e-08 | 1.51 | 3.30e-06 |
| *KCNJ15* | potassium voltage-gated channel subfamily J member 15 | 1.39 | 6.80e-06 | 1.48 | 4.10e-05 |
| *TMEM62* | transmembrane protein 62 | 1.39 | <1.0e-08 | 1.22 | 1.90e-04 |
| *DHRS9* | dehydrogenase/reductase 9 | 1.39 | 1.80e-06 | 1.47 | 1.80e-05 |
| *TLR2* | toll like receptor 2 | 1.39 | 4.40e-08 | 1.23 | 8.80e-03 |
| *ACSL1* | acyl-CoA synthetase long chain family member 1 | 1.39 | 1.20e-07 | 1.31 | 2.00e-03 |
| *PATL2* | PAT1 homolog 2 | 1.39 | <1.0e-08 | 1.19 | 2.30e-03 |
| *DAPP1* | dual adaptor of phosphotyrosine and 3-phosphoinositides 1 | 1.39 | <1.0e-08 | 1.21 | 2.70e-04 |

| | | JAHH | | ILLUMINATE | |
|---|---|---|---|---|---|
| Gene name | Gene description | Fold difference | q value | Fold difference | q value |
| *CCNL1* | cyclin L1 | 1.39 | <1.0e-08 | 1.03 | 7.70e-01 |
| *PHF11* | PHD finger protein 11 | 1.39 | <1.0e-08 | 1.31 | 1.40e-07 |
| *TRAFD1* | TRAF-type zinc finger domain containing 1 | 1.38 | <1.0e-08 | 1.30 | 4.80e-08 |
| *SRGAP2* | SLIT-ROBO Rho GTPase activating protein 2 | 1.38 | <1.0e-08 | 1.25 | 8.00e-05 |
| *TMEM140* | transmembrane protein 140 | 1.38 | <1.0e-08 | 1.36 | 5.20e-08 |
| *SP110* | SP110 nuclear body protein | 1.38 | <1.0e-08 | 1.36 | <1.0e-08 |
| *ZEB2* | zinc finger E-box binding homeobox 2 | 1.38 | <1.0e-08 | -1.02 | 8.60e-01 |
| *CLEC2B* | C-type lectin domain family 2 member B | 1.38 | <1.0e-08 | 1.19 | 3.10e-03 |
| *HIST1H1T* | histone cluster 1 H1 family member t | 1.38 | <1.0e-08 | 1.20 | 4.80e-03 |
| *LAMP3* | lysosomal associated membrane protein 3 | 1.38 | <1.0e-08 | 1.70 | <1.0e-08 |
| *CLEC7A* | C-type lectin domain containing 7A | 1.38 | <1.0e-08 | 1.19 | 9.10e-03 |
| *TRIM5* | tripartite motif containing 5 | 1.37 | <1.0e-08 | 1.36 | <1.0e-08 |
| *KIAA1109* | KIAA1109 | 1.37 | <1.0e-08 | 1.07 | 3.70e-01 |
| *HSH2D* | hematopoietic SH2 domain containing | 1.37 | <1.0e-08 | 1.16 | 1.40e-03 |
| *TOR1B* | torsin family 1 member B | 1.37 | <1.0e-08 | 1.45 | <1.0e-08 |
| *DTX3L* | deltex E3 ubiquitin ligase 3L | 1.36 | <1.0e-08 | 1.52 | <1.0e-08 |
| *HIST1H4H* | histone cluster 1 H4 family member h | 1.36 | 9.80e-08 | 1.35 | 3.90e-03 |
| *TAP2* | transporter 2, ATP binding cassette subfamily B member | 1.36 | <1.0e-08 | 1.11 | 3.80e-02 |
| *DYNLT1* | dynein light chain Tctex-type 1 | 1.36 | <1.0e-08 | 1.20 | 2.90e-04 |
| *NOD2* | nucleotide binding oligomerization domain containing 2 | 1.35 | <1.0e-08 | 1.15 | 6.80e-02 |
| *CBWD1* | COBW domain containing 1 | 1.35 | <1.0e-08 | 1.10 | 1.10e-01 |
| *PRRG4* | proline rich and Gla domain 4 | 1.35 | 1.00e-04 | 1.57 | 6.30e-06 |
| *ARRDC3* | arrestin domain containing 3 | 1.35 | <1.0e-08 | 1.17 | 1.90e-03 |
| *ERV3-1* | endogenous retrovirus group 3 member 1, envelope | 1.35 | 8.60e-07 | 1.02 | 8.50e-01 |
| *SMN2* | survival of motor neuron 2, centromeric | 1.35 | 5.60e-05 | -1.20 | 3.30e-02 |
| *VNN1* | vanin 1 | 1.35 | 7.70e-04 | 1.47 | 2.60e-03 |
| *CD44* | CD44 molecule (Indian blood group) | 1.35 | 1.40e-06 | -1.09 | 4.40e-01 |

| | | JAHH | | ILLUMINATE | |
|---|---|---|---|---|---|
| Gene name | Gene description | Fold difference | q value | Fold difference | q value |
| *TRIM25* | tripartite motif containing 25 | 1.35 | <1.0e-08 | 1.30 | <1.0e-08 |
| *JAK2* | Janus kinase 2 | 1.35 | <1.0e-08 | 1.19 | 4.30e-04 |
| *CARD16* | caspase recruitment domain family member 16 | 1.35 | 8.40e-08 | 1.40 | 9.10e-08 |
| *ANKRD22* | ankyrin repeat domain 22 | 1.34 | 1.50e-05 | 1.61 | 4.80e-06 |
| *MAP2K6* | mitogen-activated protein kinase kinase 6 | 1.34 | <1.0e-08 | 1.28 | 4.10e-04 |
| *NMI* | N-myc and STAT interactor | 1.34 | <1.0e-08 | 1.33 | 3.50e-07 |
| *ETV7* | ETS variant 7 | 1.34 | 1.40e-08 | 1.49 | 2.10e-08 |
| *RICTOR* | RPTOR independent companion of MTOR complex 2 | 1.34 | <1.0e-08 | 1.14 | 1.30e-02 |
| *RNPC3* | RNA binding region (RNP1, RRM) containing 3 | 1.34 | <1.0e-08 | -1.01 | 9.50e-01 |
| *IL18R1* | interleukin 18 receptor 1 | 1.34 | 1.60e-04 | 1.30 | 3.50e-02 |
| *APOL6* | apolipoprotein L6 | 1.33 | <1.0e-08 | 1.34 | 1.40e-08 |
| *RNF149* | ring finger protein 149 | 1.33 | <1.0e-08 | 1.06 | 5.60e-01 |
| *CHMP5* | charged multivesicular body protein 5 | 1.33 | 1.70e-08 | 1.44 | <1.0e-08 |

*The top 150 genes upregulated (ranked from high to low) based on the difference in expression between patients with systemic lupus erythematosus (SLE) from JAHH and healthy control subjects are shown. Gene name was defined using the MetaBase (Clarivate) database. Findings were compared to those from the ILLUMINATE study, analyzed using data deposited in the NCBI GEO Database (GSE88887), where an independent group of controls was compared to ILLUMINATE patients at baseline, both in terms of the rank order of genes and the fold changes detected. Statistical significance is shown as q value for patients with SLE compared to healthy controls.

**Table S2.** Baseline gene expression in SLE patients versus healthy controls, including downregulated genes with the greatest fold change from JAHH*

| | | JAHH | | ILLUMINATE | |
|---|---|---|---|---|---|
| Gene name | Gene description | Fold difference | q value | Fold difference | q value |
| *PI3* | peptidase inhibitor 3 | -1.92 | <1.0e-08 | 1.05 | 8.40e-01 |
| *RELA* | RELA proto-oncogene, NF-kB subunit | -1.92 | <1.0e-08 | 1.07 | 8.30e-02 |
| *KLRB1* | killer cell lectin like receptor B1 | -1.85 | <1.0e-08 | -1.85 | 2.30e-08 |
| *RPL26* | ribosomal protein L26 | -1.76 | <1.0e-08 | -1.22 | 1.00e-05 |
| *RPL7* | ribosomal protein L7 | -1.71 | <1.0e-08 | -1.26 | 1.00e-06 |
| *CD79A* | CD79a molecule | -1.70 | <1.0e-08 | -1.08 | 6.90e-01 |
| *TRAPPC1* | trafficking protein particle complex 1 | -1.67 | <1.0e-08 | 1.06 | 1.60e-01 |
| *CLIC3* | chloride intracellular channel 3 | -1.65 | <1.0e-08 | -1.12 | 7.30e-02 |
| *TCF7* | transcription factor 7 | -1.60 | <1.0e-08 | -1.15 | 1.70e-01 |
| *RPL6* | ribosomal protein L6 | -1.58 | <1.0e-08 | -1.22 | 9.50e-04 |
| *KLRF1* | killer cell lectin like receptor F1 | -1.58 | 2.80e-07 | -1.99 | 2.40e-07 |
| *PTPRS* | protein tyrosine phosphatase, receptor type S | -1.56 | <1.0e-08 | -1.36 | <1.0e-08 |
| *HCST* | hematopoietic cell signal transducer | -1.54 | <1.0e-08 | 1.07 | 3.70e-01 |
| *RAD23A* | RAD23 homolog A, nucleotide excision repair protein | -1.53 | <1.0e-08 | -1.10 | 1.30e-01 |
| *RPS28* | ribosomal protein S28 | -1.52 | <1.0e-08 | -1.12 | 8.40e-02 |
| *HAT1* | histone acetyltransferase 1 | -1.52 | <1.0e-08 | 1.01 | 9.00e-01 |
| *RPS27* | ribosomal protein S27 | -1.52 | <1.0e-08 | -1.11 | 2.90e-02 |
| *RPS15* | ribosomal protein S15 | -1.52 | <1.0e-08 | -1.04 | 5.30e-01 |
| *MED1* | mediator complex subunit 1 | -1.50 | <1.0e-08 | 1.05 | 3.70e-01 |
| *FCER1A* | Fc fragment of IgE receptor Ia | -1.50 | 7.60e-08 | -1.44 | 1.10e-04 |
| *KIAA1324* | KIAA1324 | -1.48 | 5.70e-07 | -1.38 | 5.10e-03 |
| *SH2D1B* | SH2 domain containing 1B | -1.47 | <1.0e-08 | -1.43 | 4.40e-04 |
| *GSTM4* | glutathione S-transferase mu 4 | -1.47 | <1.0e-08 | -1.01 | 9.40e-01 |
| *WASF2* | WAS protein family member 2 | -1.46 | <1.0e-08 | -1.06 | 2.70e-01 |
| *EIF4G1* | eukaryotic translation initiation factor 4 gamma 1 | -1.46 | <1.0e-08 | 1.02 | 7.20e-01 |
| *PSMF1* | proteasome inhibitor subunit 1 | -1.45 | <1.0e-08 | -1.21 | 1.10e-03 |
| *CD5* | CD5 molecule | -1.44 | <1.0e-08 | 1.01 | 9.00e-01 |
| *RPL4* | ribosomal protein L4 | -1.43 | <1.0e-08 | -1.27 | 5.60e-04 |
| *CLPTM1* | CLPTM1, transmembrane protein | -1.43 | <1.0e-08 | 1.01 | 7.70e-01 |
| *RBM38* | RNA binding motif protein 38 | -1.42 | 8.60e-08 | -1.12 | 2.10e-01 |
| *SLC25A39* | solute carrier family 25 member 39 | -1.42 | 2.10e-07 | -1.14 | 8.20e-02 |
| *WBP2* | WW domain binding protein 2 | -1.42 | <1.0e-08 | 1.05 | 3.70e-01 |
| *TMEM109* | transmembrane protein 109 | -1.42 | <1.0e-08 | -1.02 | 8.30e-01 |
| *CLC* | Charcot-Leyden crystal galectin | -1.42 | 1.60e-03 | -1.16 | 4.20e-01 |

| | | JAHH | | ILLUMINATE | |
|---|---|---|---|---|---|
| Gene name | Gene description | Fold difference | q value | Fold difference | q value |
| *GSPT1* | G1 to S phase transition 1 | -1.42 | 2.50e-06 | -1.17 | 3.30e-02 |
| *CBLL1* | Cbl proto-oncogene like 1 | -1.42 | <1.0e-08 | -1.04 | 5.80e-01 |
| *GMFG* | glia maturation factor gamma | -1.42 | <1.0e-08 | 1.07 | 1.30e-01 |
| *GATAD2B* | GATA zinc finger domain containing 2B | -1.41 | <1.0e-08 | -1.04 | 2.20e-01 |
| *AIF1* | allograft inflammatory factor 1 | -1.41 | <1.0e-08 | -1.04 | 4.20e-01 |
| *RPS17* | ribosomal protein S17 | -1.40 | 1.10e-06 | -1.15 | 7.20e-04 |
| *ATP6V0C* | ATPase H+ transporting V0 subunit c | -1.40 | <1.0e-08 | 1.01 | 8.70e-01 |
| *HBZ* | hemoglobin subunit zeta | -1.40 | 1.10e-04 | -1.16 | 3.70e-01 |
| *FTH1* | ferritin heavy chain 1 | -1.40 | <1.0e-08 | 1.00 | 9.70e-01 |
| *OR2W3* | olfactory receptor family 2 subfamily W member 3 | -1.39 | 7.30e-04 | -1.17 | 2.00e-01 |
| *WAS* | Wiskott-Aldrich syndrome | -1.39 | <1.0e-08 | 1.06 | 2.60e-02 |
| *SAP130* | Sin3A associated protein 130 | -1.39 | <1.0e-08 | -1.01 | 7.30e-01 |
| *MED25* | mediator complex subunit 25 | -1.39 | <1.0e-08 | 1.01 | 9.10e-01 |
| *SF3A2* | splicing factor 3a subunit 2 | -1.39 | <1.0e-08 | -1.04 | 2.90e-01 |
| *CD74* | CD74 molecule | -1.39 | <1.0e-08 | -1.01 | 9.60e-01 |
| *R3HDM4* | R3H domain containing 4 | -1.39 | 2.90e-07 | 1.07 | 3.90e-01 |
| *NRGN* | neurogranin | -1.38 | 1.90e-07 | -1.03 | 7.60e-01 |
| *TESPA1* | thymocyte expressed, positive selection associated 1 | -1.38 | 1.10e-07 | -1.10 | 3.50e-01 |
| *NFIX* | nuclear factor I X | -1.38 | <1.0e-08 | -1.09 | 1.90e-01 |
| *HIST1H4E* | histone cluster 1 H4 family member e | -1.38 | <1.0e-08 | -1.16 | 3.80e-03 |
| *ZFP36* | ZFP36 ring finger protein | -1.38 | <1.0e-08 | 1.16 | 3.10e-05 |
| *FCER2* | Fc fragment of IgE receptor II | -1.38 | <1.0e-08 | -1.18 | 5.20e-02 |
| *CD6* | CD6 molecule | -1.37 | <1.0e-08 | -1.01 | 9.00e-01 |
| *CD22* | CD22 molecule | -1.37 | 4.60e-05 | -1.21 | 1.90e-01 |
| *NOG* | noggin | -1.37 | <1.0e-08 | -1.33 | 1.30e-03 |
| *S100A12* | S100 calcium binding protein A12 | -1.37 | 1.80e-04 | 1.24 | 4.80e-02 |
| *FAM43A* | family with sequence similarity 43 member A | -1.37 | <1.0e-08 | -1.16 | 8.70e-04 |
| *GZMA* | granzyme A | -1.37 | 5.90e-04 | -1.22 | 1.10e-01 |
| *MYADM* | myeloid associated differentiation marker | -1.37 | <1.0e-08 | 1.11 | 3.70e-03 |
| *SH3BGRL3* | SH3 domain binding glutamate rich protein like 3 | -1.37 | <1.0e-08 | 1.02 | 4.50e-01 |
| *SIGLEC10* | sialic acid binding Ig like lectin 10 | -1.36 | <1.0e-08 | -1.18 | 7.50e-04 |
| *POLE3* | DNA polymerase epsilon 3, accessory subunit | -1.36 | <1.0e-08 | 1.02 | 7.50e-01 |
| *PRKCSH* | protein kinase C substrate 80K-H | -1.36 | <1.0e-08 | -1.09 | 3.80e-02 |
| *KIR3DS1* | killer cell immunoglobulin like receptor, three Ig domains and short | -1.35 | 3.30e-08 | -1.11 | 4.30e-01 |

| | | JAHH | | ILLUMINATE | |
|---|---|---|---|---|---|
| Gene name | Gene description | Fold difference | q value | Fold difference | q value |
| | cytoplasmic tail 1 | | | | |
| *CTSW* | cathepsin W | -1.35 | 3.10e-07 | -1.10 | 3.20e-01 |
| *CD27* | CD27 molecule | -1.35 | <1.0e-08 | -1.12 | 2.30e-01 |
| *DAAM1* | dishevelled associated activator of morphogenesis 1 | -1.35 | <1.0e-08 | -1.05 | 4.00e-01 |
| *ALG3* | ALG3, alpha-1,3-mannosyltransferase | -1.35 | <1.0e-08 | 1.12 | 2.80e-02 |
| *RPL17* | ribosomal protein L17 | -1.35 | 4.30e-05 | -1.16 | 2.50e-03 |
| *FXYD5* | FXYD domain containing ion transport regulator 5 | -1.35 | <1.0e-08 | -1.00 | 9.60e-01 |
| *SLC4A1* | solute carrier family 4 member 1 (Diego blood group) | -1.34 | 2.00e-03 | -1.19 | 2.20e-01 |
| *ARHGAP1* | Rho GTPase activating protein 1 | -1.34 | <1.0e-08 | 1.01 | 9.10e-01 |
| *NDUFS5* | NADH:ubiquinone oxidoreductase subunit S5 | -1.34 | <1.0e-08 | -1.11 | 7.10e-03 |
| *CCR7* | C-C motif chemokine receptor 7 | -1.34 | 5.30e-05 | -1.16 | 2.60e-01 |
| *SEPT1* | septin 1 | -1.34 | <1.0e-08 | -1.08 | 2.70e-01 |
| *NR1D1* | nuclear receptor subfamily 1 group D member 1 | -1.34 | <1.0e-08 | -1.02 | 7.40e-01 |
| *PPP2R1A* | protein phosphatase 2 scaffold subunit Aalpha | -1.34 | <1.0e-08 | 1.01 | 9.00e-01 |
| *KIR2DL1* | killer cell immunoglobulin like receptor, two Ig domains and long cytoplasmic tail 1 | -1.33 | <1.0e-08 | -1.11 | 3.20e-01 |
| *PHB2* | prohibitin 2 | -1.33 | <1.0e-08 | -1.06 | 2.60e-01 |
| *IL2RB* | interleukin 2 receptor subunit beta | -1.33 | <1.0e-08 | -1.14 | 2.80e-03 |
| *GUCD1* | guanylyl cyclase domain containing 1 | -1.33 | <1.0e-08 | -1.11 | 9.60e-02 |
| *SLC29A3* | solute carrier family 29 member 3 | -1.33 | <1.0e-08 | 1.02 | 7.10e-01 |
| *PFN1* | profilin 1 | -1.33 | <1.0e-08 | 1.05 | 2.70e-01 |
| *CS* | citrate synthase | -1.33 | <1.0e-08 | -1.11 | 1.60e-02 |
| *GATA3* | GATA binding protein 3 | -1.33 | <1.0e-08 | -1.05 | 4.40e-01 |
| *FECH* | ferrochelatase | -1.33 | 2.70e-04 | -1.36 | 4.20e-04 |
| *SF3B4* | splicing factor 3b subunit 4 | -1.33 | <1.0e-08 | 1.02 | 2.70e-01 |
| *FLT3LG* | fms related tyrosine kinase 3 ligand | -1.32 | <1.0e-08 | -1.02 | 8.50e-01 |
| *PCIF1* | PDX1 C-terminal inhibiting factor 1 | -1.32 | <1.0e-08 | -1.01 | 8.60e-01 |
| *SCAMP2* | secretory carrier membrane protein 2 | -1.32 | <1.0e-08 | 1.04 | 2.60e-01 |
| *EPB42* | erythrocyte membrane protein band 4.2 | -1.32 | 2.30e-03 | -1.29 | 5.80e-02 |
| *TPP1* | tripeptidyl peptidase 1 | -1.32 | <1.0e-08 | 1.09 | 1.50e-02 |
| *PRAF2* | PRA1 domain family member 2 | -1.32 | <1.0e-08 | 1.09 | 4.60e-05 |
| *PRPF8* | pre-mRNA processing factor 8 | -1.32 | <1.0e-08 | -1.11 | 7.10e-02 |
| *RAC2* | Rac family small GTPase 2 | -1.32 | <1.0e-08 | 1.07 | 1.90e-02 |

| | | JAHH | | ILLUMINATE | |
|---|---|---|---|---|---|
| Gene name | Gene description | Fold difference | q value | Fold difference | q value |
| *ZNF107* | zinc finger protein 107 | -1.32 | 5.60e-06 | 1.02 | 8.90e-01 |
| *APEX1* | apurinic/apyrimidinic endodeoxyribonuclease 1 | -1.32 | <1.0e-08 | -1.18 | 8.90e-03 |
| *FLOT1* | flotillin 1 | -1.32 | <1.0e-08 | 1.09 | 1.60e-01 |
| *HEMGN* | hemogen | -1.32 | 3.50e-05 | -1.32 | 1.40e-04 |
| *HCFC1* | host cell factor C1 | -1.32 | <1.0e-08 | 1.01 | 8.10e-01 |
| *FHL3* | four and a half LIM domains 3 | -1.32 | <1.0e-08 | 1.03 | 6.20e-01 |
| *CHERP* | calcium homeostasis endoplasmic reticulum protein | -1.32 | <1.0e-08 | 1.03 | 1.70e-01 |
| *SELENBP1* | selenium binding protein 1 | -1.32 | 2.80e-04 | -1.13 | 3.00e-01 |
| *EPB41* | erythrocyte membrane protein band 4.1 | -1.31 | 4.90e-08 | -1.22 | 1.90e-04 |
| *CITED2* | Cbp/p300 interacting transactivator with Glu/Asp rich carboxy-terminal domain 2 | -1.31 | <1.0e-08 | 1.05 | 1.50e-01 |
| *SLC9A3R1* | SLC9A3 regulator 1 | -1.31 | <1.0e-08 | 1.01 | 7.90e-01 |
| *PPBP* | pro-platelet basic protein | -1.31 | 7.30e-03 | -1.40 | 9.70e-04 |
| *RSL1D1* | ribosomal L1 domain containing 1 | -1.31 | 1.10e-08 | -1.11 | 2.10e-01 |
| *SERTAD2* | SERTA domain containing 2 | -1.31 | <1.0e-08 | -1.02 | 6.20e-01 |
| *PRKACA* | protein kinase cAMP-activated catalytic subunit alpha | -1.31 | <1.0e-08 | -1.05 | 5.00e-02 |
| *NFATC2* | nuclear factor of activated T cells 2 | -1.31 | <1.0e-08 | -1.10 | 1.40e-01 |
| *KIR3DL2* | killer cell immunoglobulin like receptor, three Ig domains and long cytoplasmic tail 2 | -1.31 | <1.0e-08 | -1.08 | 4.60e-01 |
| *GYPC* | glycophorin C (Gerbich blood group) | -1.31 | <1.0e-08 | -1.14 | 2.50e-02 |
| *CAT* | catalase | -1.31 | 7.30e-08 | -1.19 | 2.60e-03 |
| *PLP2* | proteolipid protein 2 | -1.31 | <1.0e-08 | 1.17 | 5.70e-05 |
| *SF3A1* | splicing factor 3a subunit 1 | -1.31 | <1.0e-08 | 1.01 | 7.10e-01 |
| *SIRT2* | sirtuin 2 | -1.31 | <1.0e-08 | 1.03 | 4.90e-01 |
| *SLC6A8* | solute carrier family 6 member 8 | -1.31 | 7.70e-05 | -1.03 | 8.20e-01 |
| *MLXIP* | MLX interacting protein | -1.31 | <1.0e-08 | -1.05 | 1.40e-01 |
| *TMEM238* | transmembrane protein 238 | -1.31 | <1.0e-08 | 1.08 | 5.10e-02 |
| *PHOSPHO1* | phosphoethanolamine/phosphocholine phosphatase | -1.31 | 1.80e-06 | -1.02 | 8.50e-01 |
| *KLF2* | Kruppel like factor 2 | -1.31 | <1.0e-08 | 1.01 | 8.50e-01 |
| *KRT1* | keratin 1 | -1.30 | <1.0e-08 | -1.13 | 6.70e-02 |
| *SRM* | spermidine synthase | -1.30 | <1.0e-08 | 1.06 | 2.80e-01 |
| *SIGLEC9* | sialic acid binding Ig like lectin 9 | -1.30 | <1.0e-08 | 1.08 | 2.20e-01 |
| *MAF1* | MAF1 homolog, negative regulator of RNA polymerase III | -1.30 | <1.0e-08 | -1.05 | 3.60e-01 |
| *ZFP36L2* | ZFP36 ring finger protein like 2 | -1.30 | <1.0e-08 | -1.03 | 6.50e-01 |

| | | JAHH | | ILLUMINATE | |
|---|---|---|---|---|---|
| Gene name | Gene description | Fold difference | q value | Fold difference | q value |
| *GSK3A* | glycogen synthase kinase 3 alpha | -1.30 | <1.0e-08 | 1.03 | 4.00e-01 |
| *HLA-DRA* | major histocompatibility complex, class II, DR alpha | -1.30 | 1.40e-06 | -1.09 | 4.10e-01 |
| *POM121C* | POM121 transmembrane nucleoporin C | -1.30 | <1.0e-08 | -1.09 | 1.20e-03 |
| *DCAF12* | DDB1 and CUL4 associated factor 12 | -1.30 | 8.50e-05 | -1.11 | 1.30e-01 |
| *RAB1B* | RAB1B, member RAS oncogene family | -1.30 | <1.0e-08 | 1.04 | 9.70E-02 |
| *BANF1* | barrier to autointegration factor 1 | -1.30 | <1.0e-08 | 1.04 | 2.50e-01 |
| *OGFOD1* | 2-oxoglutarate and iron dependent oxygenase domain containing 1 | -1.30 | <1.0e-08 | 1.01 | 9.40e-01 |
| *ANXA1* | annexin A1 | -1.30 | 1.80e-05 | 1.01 | 8.80e-01 |
| *PTBP1* | polypyrimidine tract binding protein 1 | -1.30 | <1.0e-08 | -1.03 | 6.30e-01 |
| *KIR2DL3* | killer cell immunoglobulin like receptor, two Ig domains and long cytoplasmic tail 3 | -1.30 | 2.30e-07 | -1.08 | 5.70e-01 |
| *COPE* | coatomer protein complex subunit epsilon | -1.30 | <1.0e-08 | 1.12 | 4.70e-04 |
| *CHI3L1* | chitinase 3 like 1 | -1.30 | 9.00e-04 | -1.17 | 2.40e-01 |
| *SP2* | Sp2 transcription factor | -1.30 | <1.0e-08 | 1.06 | 4.10e-04 |
| *KIR2DS4* | killer cell immunoglobulin like receptor, two Ig domains and short cytoplasmic tail 4 | -1.30 | 2.10e-05 | 1.03 | 8.80e-01 |
| *ZHX2* | zinc fingers and homeoboxes 2 | -1.30 | <1.0e-08 | -1.06 | 9.20e-02 |
| *BSG* | basigin (Ok blood group) | -1.30 | 1.10e-05 | -1.12 | 1.40e-01 |
| *ZNF385A* | zinc finger protein 385A | -1.30 | <1.0e-08 | 1.05 | 3.40e-01 |
| *ACTN4* | actinin alpha 4 | -1.29 | <1.0e-08 | 1.02 | 6.30e-01 |
| *FGFBP2* | fibroblast growth factor binding protein 2 | -1.29 | 5.30e-05 | -1.19 | 1.50e-01 |

*The top 150 genes downregulated (ranked from high to low) based on the difference in expression between patients with systemic lupus erythematosus (SLE) from JAHH and healthy control subjects are shown. Gene name was defined using the MetaBase (Clarivate) database. Findings were compared to those from the ILLUMINATE study, analyzed using data deposited in the NCBI GEO Database (GSE88887), where an independent group of controls was compared to ILLUMINATE patients at baseline, both in terms of the rank order of genes and the fold

changes detected. Statistical significance is shown as q value for patients with SLE compared to healthy controls.

**Table S3.** Pharmacologically-induced changes in gene expression in response to baricitinib treatment*

| | | Baricitinib 4-mg | | | | | |
|---|---|---|---|---|---|---|---|
| | | Baseline to Week 4 | | Baseline to Week 12 | | Baseline to Week 24 | |
| Gene name | Gene description | Fold difference | q value | Fold difference | q value | Fold difference | q value |
| *IFI44L* | interferon induced protein 44 like | -1.18 | 4.0e-01 | -1.50 | 3.1e-03 | -1.32 | 1.2e-01 |
| *RSAD2* | radical S-adenosyl methionine domain containing 2 | -1.15 | 4.5e-01 | -1.48 | 2.9e-03 | -1.25 | 1.6e-01 |
| *IFI44* | interferon induced protein 44 | -1.11 | 6.4e-01 | -1.38 | 4.1e-03 | -1.21 | 2.0e-01 |
| *IFI27* | interferon alpha inducible protein 27 | -1.59 | 2.0e-03 | -1.63 | 2.9e-03 | -1.32 | 1.6e-01 |
| *IFIT1* | interferon induced protein with tetratricopeptide repeats 1 | -1.16 | 3.8e-01 | -1.43 | 2.9e-03 | -1.23 | 1.6e-01 |
| *MX1* | MX dynamin like GTPase 1 | -1.11 | 4.9e-01 | -1.35 | 3.2e-03 | -1.22 | 1.2e-01 |
| *HERC5* | HECT and RLD domain containing E3 ubiquitin protein ligase 5 | -1.09 | 6.4e-01 | -1.29 | 8.2e-03 | -1.16 | 2.1e-01 |
| *EPSTI1* | epithelial stromal interaction 1 | -1.03 | 9.3e-01 | -1.21 | 2.5e-02 | -1.13 | 2.3e-01 |
| *OAS3* | 2'-5'-oligoadenylate synthetase 3 | -1.13 | 3.0e-01 | -1.29 | 3.1e-03 | -1.18 | 1.3e-01 |
| *DDX60* | DExD/H-box helicase 60 | -1.01 | 9.8e-01 | -1.21 | 2.8e-02 | -1.11 | 3.2e-01 |
| *CMPK2* | cytidine/uridine monophosphate kinase 2 | -1.13 | 2.4e-01 | -1.23 | 3.9e-03 | -1.16 | 1.2e-01 |
| *XAF1* | XIAP associated factor 1 | -1.07 | 4.9e-01 | -1.22 | 3.1e-03 | -1.14 | 1.3e-01 |
| *IFIT3* | interferon induced protein with tetratricopeptide repeats 3 | -1.05 | 7.8e-01 | -1.26 | 3.9e-03 | -1.11 | 3.1e-01 |
| *OAS1* | 2'-5'-oligoadenylate synthetase 1 | -1.12 | 2.4e-01 | -1.25 | 2.9e-03 | -1.16 | 1.2e-01 |
| *EIF2AK2* | eukaryotic translation initiation factor 2 alpha kinase 2 | -1.05 | 7.8e-01 | -1.18 | 3.6e-02 | -1.10 | 3.2e-01 |
| *PLSCR1* | phospholipid scramblase 1 | -1.04 | 7.8e-01 | -1.19 | 2.5e-02 | -1.07 | 4.8e-01 |
| *USP18* | ubiquitin specific peptidase 18 | -1.15 | 1.1e-01 | -1.27 | 2.3e-03 | -1.20 | 7.6e-02 |
| *IFIT2* | interferon induced protein with tetratricopeptide repeats 2 | -1.05 | 7.8e-01 | -1.24 | 4.1e-03 | -1.11 | 2.8e-01 |
| *IFIT5* | interferon induced protein with tetratricopeptide repeats 5 | -1.04 | 8.5e-01 | -1.22 | 8.2e-03 | -1.07 | 4.2e-01 |

| | | Baricitinib 4-mg | | | | | |
|---|---|---|---|---|---|---|---|
| | | _Baseline to Week 4_ | | _Baseline to Week 12_ | | _Baseline to Week 24_ | |
| Gene name | Gene description | Fold difference | q value | Fold difference | q value | Fold difference | q value |
| *OAS2* | 2'-5'-oligoadenylate synthetase 2 | -1.15 | 3.0e-01 | -1.26 | 8.0e-03 | -1.24 | 7.6e-02 |
| *SAMD9L* | sterile alpha motif domain containing 9 like | -1.00 | 9.9e-01 | -1.14 | 9.1e-02 | -1.08 | 4.2e-01 |
| *SERPING1* | serpin family G member 1 | 1.02 | 9.8e-01 | -1.06 | 5.8e-01 | -1.07 | 5.9e-01 |
| *IFI6* | interferon alpha inducible protein 6 | -1.15 | 3.0e-01 | -1.38 | 2.8e-03 | -1.21 | 1.2e-01 |
| *IFIH1* | interferon induced with helicase C domain 1 | -1.01 | 9.8e-01 | -1.19 | 2.8e-02 | -1.09 | 4.1e-01 |
| *DDX58* | DExD/H-box helicase 58 | 1.01 | 9.8e-01 | -1.14 | 5.4e-02 | -1.06 | 4.9e-01 |
| *ZCCHC2* | zinc finger CCHC-type containing 2 | -1.04 | 7.8e-01 | -1.13 | 5.4e-02 | -1.10 | 2.3e-01 |
| *USP41* | ubiquitin specific peptidase 41 | -1.12 | 1.1e-01 | -1.20 | 2.9e-03 | -1.16 | 7.6e-02 |
| *OASL* | 2'-5'-oligoadenylate synthetase like | -1.14 | 1.2e-01 | -1.22 | 2.9e-03 | -1.17 | 7.6e-02 |
| *PARP12* | poly(ADP-ribose) polymerase family member 12 | -1.04 | 6.4e-01 | -1.15 | 8.4e-03 | -1.09 | 2.1e-01 |
| *DDX60L* | DExD/H-box 60 like | -1.00 | 9.8e-01 | -1.10 | 1.2e-01 | -1.07 | 4.2e-01 |
| *CARD17* | caspase recruitment domain family member 17 | 1.03 | 9.4e-01 | -1.07 | 3.9e-01 | -1.07 | 4.9e-01 |
| *PARP14* | poly(ADP-ribose) polymerase family member 14 | 1.02 | 9.4e-01 | -1.11 | 1.3e-01 | -1.07 | 4.2e-01 |
| *RTP4* | receptor transporter protein 4 | -1.08 | 4.5e-01 | -1.12 | 9.1e-02 | -1.09 | 3.0e-01 |
| *GBP1* | guanylate binding protein 1 | 1.07 | 6.4e-01 | -1.04 | 6.2e-01 | -1.04 | 6.1e-01 |
| *OR56B1* | olfactory receptor family 56 subfamily B member 1 | -1.00 | 9.8e-01 | -1.12 | 2.5e-02 | -1.07 | 3.2e-01 |
| *STAT1* | signal transducer and activator of transcription 1 | 1.02 | 9.4e-01 | -1.07 | 2.7e-01 | -1.06 | 4.2e-01 |
| *TRIM22* | tripartite motif containing 22 | 1.04 | 7.4e-01 | -1.09 | 6.3e-02 | -1.03 | 5.8e-01 |
| *HERC6* | HECT and RLD domain containing E3 ubiquitin protein ligase family member 6 | -1.10 | 2.7e-01 | -1.17 | 6.1e-03 | -1.15 | 7.6e-02 |
| *PARP9* | poly(ADP-ribose) polymerase family | -1.01 | 9.8e-01 | -1.09 | 1.2e-01 | -1.06 | 4.2e-01 |

| | | Baricitinib 4-mg | | | | | |
|---|---|---|---|---|---|---|---|
| | | _Baseline to Week 4_ | | _Baseline to Week 12_ | | _Baseline to Week 24_ | |
| Gene name | Gene description | Fold difference | q value | Fold difference | q value | Fold difference | q value |
| | member 9 | | | | | | |
| *SPATS2L* | spermatogenesis associated serine rich 2 like | -1.07 | 3.0e-01 | -1.10 | 4.9e-02 | -1.08 | 2.1e-01 |
| *GBP5* | guanylate binding protein 5 | 1.13 | 3.0e-01 | 1.02 | 7.8e-01 | -1.01 | 8.9e-01 |
| *STAT2* | signal transducer and activator of transcription 2 | -1.00 | 9.8e-01 | -1.07 | 2.0e-01 | -1.05 | 4.5e-01 |
| *FCGR1A* | Fc fragment of IgG receptor Ia | 1.05 | 7.8e-01 | -1.05 | 5.8e-01 | -1.03 | 7.7e-01 |
| *LAP3* | leucine aminopeptidase 3 | -1.00 | 9.8e-01 | -1.13 | 2.8e-02 | -1.05 | 4.8e-01 |
| *DOCK4* | dedicator of cytokinesis 4 | 1.09 | 6.4e-01 | 1.13 | 1.8e-01 | -1.02 | 8.1e-01 |
| *CD274* | CD274 molecule | 1.11 | 3.0e-01 | 1.04 | 5.8e-01 | 1.06 | 4.8e-01 |
| *LY6E* | lymphocyte antigen 6 family member E | -1.18 | 7.0e-02 | -1.22 | 2.9e-03 | -1.17 | 7.6e-02 |
| *TNFAIP6* | TNF alpha induced protein 6 | 1.02 | 9.4e-01 | -1.07 | 3.6e-01 | 1.05 | 5.8e-01 |
| *PNPT1* | polyribonucleotide nucleotidyltransferase 1 | -1.02 | 9.4e-01 | -1.14 | 5.4e-02 | -1.05 | 5.3e-01 |
| *PANK2* | pantothenate kinase 2 | 1.07 | 6.4e-01 | -1.12 | 1.3e-01 | 1.01 | 9.1e-01 |

*The top 50 genes upregulated (ranked from high to low) based on the difference in expression between patients with systemic lupus erythematosus (SLE) and healthy control subjects are represented with changes from baseline in gene expression for baricitinib 4-mg at weeks 4, 12, and 24 shown. Gene name was defined using the MetaBase (Clarivate) database. Statistical significance is shown as q value for baricitinib 4-mg compared to placebo.

**Table S4.** Number of STAT related genes whose variation have statistically significant association to SLEDAI-2K variation*

| STAT | Week | Number of downstream genes | Number of downstream genes with FDR $p < 0.1$ | Number of downstream genes with $p < 0.05$ |
|---|---|---|---|---|
| *STAT1* | 2 | 135 | 30 | 45 |
| *STAT1* | 4 | 135 | 0 | 5 |
| *STAT1* | 12 | 135 | 0 | 2 |
| *STAT1* | 24 | 135 | 0 | 1 |
| *STAT2* | 2 | 38 | 11 | 13 |
| *STAT2* | 4 | 38 | 0 | 2 |
| *STAT2* | 12 | 38 | 0 | 0 |
| *STAT2* | 24 | 38 | 0 | 0 |
| *STAT4* | 2 | 6 | 0 | 1 |
| *STAT4* | 4 | 6 | 0 | 0 |
| *STAT4* | 12 | 6 | 0 | 0 |
| *STAT4* | 24 | 6 | 0 | 0 |

*Spearman rank coefficient correlations between variation in Systemic Lupus Erythematosus Disease Activity Index-2000 (SLEDAI-2K) (from baseline) and variation in log2 expression (from baseline) of genes were computed at weeks 2, 4, 12, and 24 with baricitinib 4-mg treatment and tested for statistically significant differences from zero. The genes analyzed were selected from the MetaCore Database for those genes which are downstream of *STAT1*, *STAT2*, or *STAT4*. The number of selected downstream genes for each signal transducer and activator of transcription (STAT) is shown, with the number of statistically significant genes shown according to two different criteria: unadjusted $p < 0.05$ or false discovery rate (FDR) (computed within each STAT and time point) $p < 0.1$. Most of the significant changes occurred at the earliest time point measured, week 2. *STAT1* had the largest number of downstream genes and also the largest number of significantly associated genes according to both criteria.
